# Not All ^3^MC States Are the Same: The Role of ^3^MC_cis_ States in the Photochemical N^∧^N Ligand Release from [Ru(bpy)_2_(N^∧^N)]^2+^ Complexes

**DOI:** 10.1021/acs.inorgchem.2c03146

**Published:** 2022-11-30

**Authors:** Katie Eastham, Paul A. Scattergood, Danny Chu, Rayhaan Z. Boota, Adrien Soupart, Fabienne Alary, Isabelle M. Dixon, Craig R. Rice, Samantha J. O. Hardman, Paul I. P. Elliott

**Affiliations:** † Department of Chemistry, University of Huddersfield, Queensgate, Huddersfield HD1 3DH, U.K.; ‡ Centre for Functional Materials, University of Huddersfield, Queensgate, Huddersfield HD1 3DH, U.K.; § Manchester Institute of Biotechnology, The University of Manchester, 131 Princess Street, Manchester M1 7DN, U.K.; ∥ Laboratoire de Chimie et Physique Quantiques, UMR 5626 CNRS/Université Toulouse 3—Paul Sabatier, Université de Toulouse, 118 route de Narbonne, Toulouse 31062, France

## Abstract

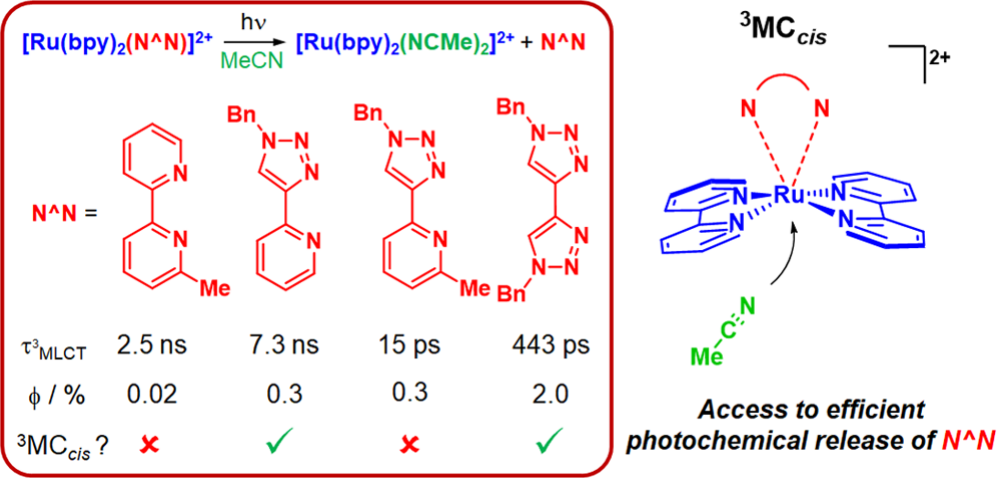

Ruthenium(II) complexes feature prominently in the development of agents for photoactivated chemotherapy; however, the excited-state mechanisms by which photochemical ligand release operates remain unclear. We report here a systematic experimental and computational study of a series of complexes [Ru(bpy)_2_(N^∧^N)]^2+^ (bpy = 2,2′-bipyridyl; N^∧^N = bpy (**1**), 6-methyl-2,2′-bipyridyl (**2**), 6,6′-dimethyl-2,2′-bipyridyl (**3**), 1-benzyl-4-(pyrid-2-yl)-1,2,3-triazole (**4**), 1-benzyl-4-(6-methylpyrid-2-yl)-1,2,3-triazole (**5**), 1,1′-dibenzyl-4,4′-bi-1,2,3-triazolyl (**6**)), in which we probe the contribution to the promotion of photochemical N^∧^N ligand release of the introduction of sterically encumbering methyl substituents and the electronic effect of replacement of pyridine by 1,2,3-triazole donors in the N^∧^N ligand. Complexes **2** to **6** all release the ligand N^∧^N on irradiation in acetonitrile solution to yield *cis-*[Ru(bpy)_2_(NCMe)_2_]^2+^, with resultant photorelease quantum yields that at first seem counter-intuitive and span a broad range. The data show that incorporation of a single sterically encumbering methyl substituent on the N^∧^N ligand (**2** and **5**) leads to a significantly enhanced rate of triplet metal-to-ligand charge-transfer (^3^MLCT) state deactivation but with little promotion of photoreactivity, whereas replacement of pyridine by triazole donors (**4** and **6**) leads to a similar rate of ^3^MLCT deactivation but with much greater photochemical reactivity. The data reported here, discussed in conjunction with previously reported data on related complexes, suggest that monomethylation in **2** and **5** sterically inhibits the formation of a ^3^MC_cis_ state but promotes the population of ^3^MC_trans_ states which rapidly deactivate ^3^MLCT states and are prone to mediating ground-state recovery. On the other hand, increased photochemical reactivity in **4** and **6** seems to stem from the accessibility of ^3^MC_cis_ states. The data provide important insights into the excited-state mechanism of photochemical ligand release by Ru(II) tris-bidentate complexes.

## Abstract 2

Structure−property relationships are explored, which govern the efficiency of photochemical ligand release. Results reveal that while steric strain may stabilize and promote the population of ^3^MC states in general, this does not necessarily lead to promotion of photochemistry. The data suggest that different classes of ^3^MC state play differing roles in relation to ground-state recovery versus promotion of photochemistry and that ^3^MC_cis_ states play a key role in photochemical ligand release.

## Introduction

The photochemistry of ruthenium(II) complexes is a current subject of some prominence in the literature due to their potential application in photoactivated chemotherapy (PACT).^1−4^ Here, a nontoxic ruthenium(II) complex that is photochemically labile undergoes photorelease of a ligand,^5,6^ yielding cytotoxic metal-containing and/or ligand fragments^7,8^ and enables excellent spatial and temporal control of drug release and anticancer activity. Further, PACT does not rely on the presence of molecular oxygen required for photodynamic therapy (PDT)^9,10^ and therefore has advantages under the hypoxic conditions found in tumors.^11^

**Figure 1:**
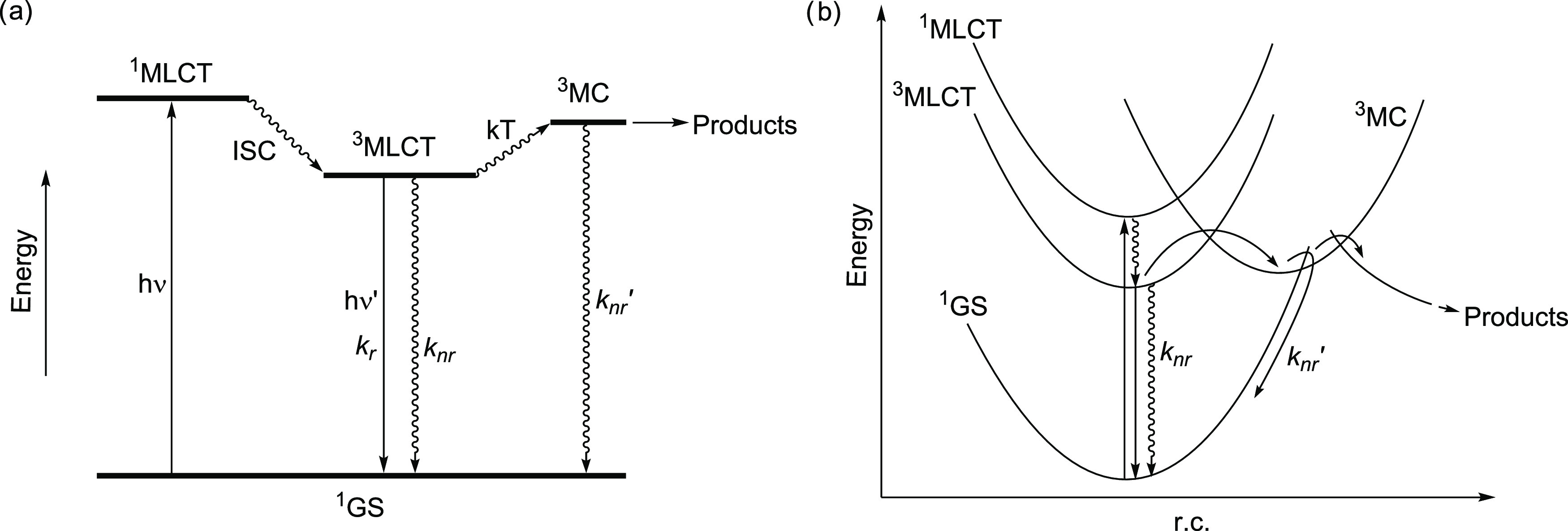
Qualitative Jablonski (a) and potential energy surface (b) diagrams depicting photophysical and photochemical processes for a [Ru(bpy)_3_]^2+^-like complex.

Key to achieving efficient photochemical reactivity in complexes of this class is the accessibility of photoreactive triplet metal-centered (^3^MC) states (associated with the population of Ru–N antibonding dσ* orbitals) via thermal population from triplet metal-to-ligand charge-transfer (^3^MLCT) states,^12,13^ themselves resulting from intersystem crossing from photoexcited singlet MLCT states (Figure 1a).^14,15^ Due to the population of antibonding orbitals, ^3^MC states experience distortions and metal–ligand bond elongations with potential energy surface (PES) minima that are significantly displaced relative to minima for the ground- and MLCT states (Figure 1b).^16−18^ This can therefore result in not only rapid nonradiative decay to the ground state^19,20^ but also ligand dissociation and the formation of photoproducts.^21^

The archetypal complex [Ru(bpy)_3_]^2+^ (bpy = 2,2′-bipyridyl)^22^ exhibits efficient luminescence from the ^3^MLCT state^21^ and is well known to undergo photoanation with the release of a bpy ligand^23−27^ with realization of selective photochemical ligand release possible in heteroleptic complexes.^28,29^ The efficiency of selective photochemical ligand release can be significantly enhanced in sterically encumbered derivatives featuring weakened Ru–N bonds.^30^ This stabilizes the ^3^MC state relative to the ^3^MLCT state, which promotes efficient photochemical ligand release. For example, the complex [Ru(bpy)_2_(dmbpy)]^2+^ (dmbpy = 6,6′-dimethyl-2,2′-bipyridyl) is not luminescent in room temperature (r.t.) solution and readily releases the dmbpy ligand upon irradiation in donor solvents.^31^

**Scheme 1:**
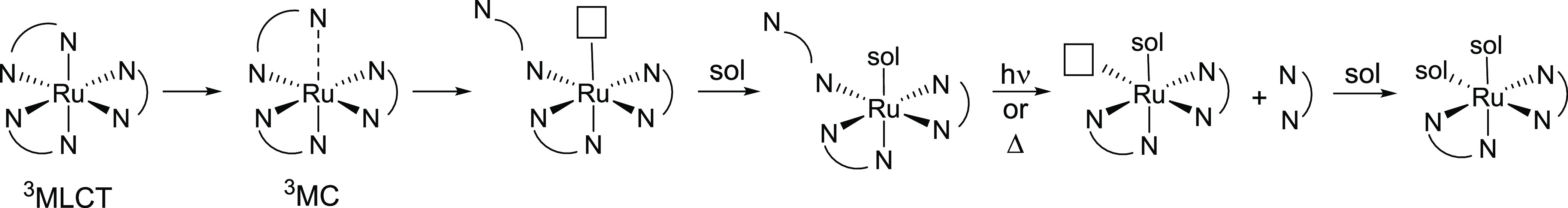
Historically Accepted Mechanism for Photochemical Ligand Release from [Ru(bpy)_3_]^2+^-Type Complexes sol = coordinating solvent ligand.

The historically accepted mechanism for photoinitiated ligand release from trischelate complexes (Scheme 1)^21,23,26,27,32^ involves a population of thermally accessible ^3^MC states from the ^3^MLCT state, which can go on to facilitate either ground-state recovery or ligand dechelation. Subsequent solvent trapping of the coordinatively unsaturated species yields a ligand-loss intermediate with a monodentate N^∧^N ligand.^23−25^ A secondary photochemical or thermally driven process then results in the formal loss of the monodentate N^∧^N ligand and the formation of the final bis-solvento product complex. However, commonly invoked ligand-loss intermediates from tris(diimine) complexes are rarely observed.^33−37^ Further, the exact nature of the processes occurring on the triplet excited-state potential energy surface has not been well understood, in particular, the exact geometric and electronic character of the ^3^MC states in question. ^3^MC states are generally spectroscopically dark and therefore intrinsically difficult to study.^38,39^

Computational studies have provided highly illuminating results in this regard, which have shed light on the nature of these important excited states. Numerous reports have detailed the pivotal role of ^3^MC states in mediating photochromic^40−42^ and photoracemization^43,44^ reactions of metal complexes and photochemical ligand substitution reactivity.^45−50^ These studies reveal that metal complexes can access a number of ^3^MC states of differing geometric and electronic character.^51^ Further, investigations have suggested that structural modification of complexes can modulate the relative accessibility of different ^3^MC states, states which may have preferential roles in either promoting ground-state recovery or ligand substitution, thus modulating the photochemical ligand substitution efficiency.^52^ In our studies of the coordination chemistry of 1,2,3-triazole-based ligands^53,54^ and the photophysics and photoreactivity of their resultant complexes,^36,37,55−58^ we were able to identify differing structural classes of hexacoordinate ^3^MC states, ^3^MC_trans_, and ^3^MC_cis_, where trans and cis denote the relative regiochemistry of the elongated Ru–N bonds.^51,59,60^ These latter ^3^MC_cis_ states were shown to be crucial in the observed photochemistry of Ru(II) bis(bitriazolyl) complexes.^59^

From these studies, what seems clear is that the lowest triplet state potential energy surface of complexes of this type is a highly complex landscape comprising local minima for many ^3^MC states of different geometric character (e.g., relative stereochemistry of elongated metal–ligand bonds) and denticity (hexacoordinate versus pentacoordinate, etc.). It is likely that many possible routes to photochemical ligand release exist through this excited-state potential energy landscape, which then represents different photochemical mechanistic regimes. Structure–property relationships then determine which ^3^MC states are preferentially accessible in this landscape and so which route, or routes will dominate for a particular complex and govern the efficiencies of ligand photorelease and ground-state recovery.

In this contribution, we explore the relative importance of ^3^MC state-stabilizing steric effects and ^3^MLCT state-destabilizing electronic effects on the ordering and energy gap between these states and on promoting photochemical reactivity of complexes of the form [Ru(bpy)_2_(N^∧^N)]^2+^ (where N^∧^N is a bipyridyl-, pyridyltriazole- or bitriazolyl-based ligand). We show that both inclusion of a sterically encumbering methyl group or a triazole donor in the N^∧^N ligand results in rapid deactivation of the ^3^MLCT state. However, incorporation of a nonsterically demanding triazole donor surprisingly leads to a 10-fold higher photochemical quantum yield for N^∧^N ligand release. Our results suggest that structure–property relationships exist, governing the type of ^3^MC states that are accessible from the ^3^MLCT state. The data reported here, combined with data from other recent studies, provide a compelling case for a pivotal role that ^3^MC_cis_ states^60^ play in mediating photochemical ligand release in [Ru(N^∧^N)_3_]^2+^-type complexes, offering significant insight into the photoreactive mechanistic regimes in which photochemistry for these complexes operate. In particular, the possible involvement of ^3^MC_cis_ states in mediating photochemical ligand release and solvent coordination in a single step without the need for a [Ru(bpy)_2_(κ^1^-N^∧^N)(solvent)]^2+^-type intermediate is discussed.

## Results and Discussion

**Figure 2:**
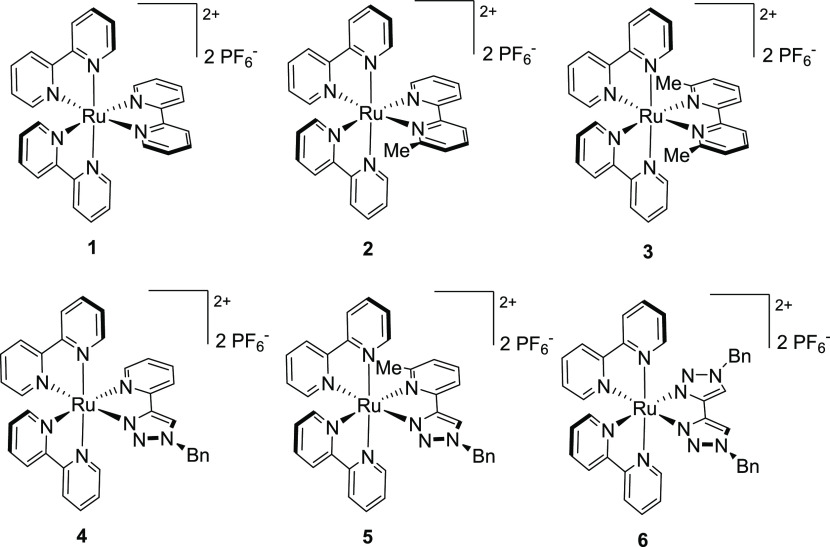
Structures of the complexes [Ru(bpy)_2_(N^∧^N)](PF_6_)_2_**1** to **6** (N^∧^N = bpy (**1**); mbpy (**2**);^64^ dmbpy (**3**);^31^ pytz (**4**);^65^ mpytz (**5**); and btz (**6**)^58^).

The complexes included in this study are depicted in Figure 2. The tris-bpy-based complexes **2** and **3** feature increasing steric congestion adjacent to the coordinating N-atoms of one bpy-based ligand to stabilize their ^3^MC states relative to their ^3^MLCT states when compared to the unencumbered complex **1**. Complexes **4** and **6** feature a pyridyltriazole and btz ligand, respectively, included to destabilize the ^3^MLCT state relative to their ^3^MC states when compared to **1**.^59^ 1,2,3-Triazoles have been shown by Sarkar and co-workers to be slightly weaker overall donors relative to pyridine based on infrared spectra of Re(I) carbonyl complexes though weaker π-acceptors based on electrochemical reduction potentials.^61^ Other studies have indicated monodentate triazole and pyridine ligands can be broadly comparable as donors.^62,63^ The smaller ring size for a triazole donor and the absence of a C–H proton adjacent to the coordinating N-atom, which is present for pyridine, makes the triazole donor less sterically demanding.

The new complex **5** featuring the mpytz ligand incorporates both a ^3^MLCT state-destabilizing triazole moiety as well as a ^3^MC state-stabilizing methyl substituent on the pyridine ring and thus might be expected to display the smallest ^3^MLCT–^3^MC gap (or indeed inverted ordering relative to **1**, **2**, **4** and **6**). The mpytz ligand was prepared in a two-step procedure through initial Sonogashira ethynylation of 2-bromo-6-methylpyridine, followed by copper(I)-catalyzed alkyne–azide cycloaddition with benzyl azide. Subsequent reaction with [Ru(bpy)_2_Cl_2_] then resulted in the formation and isolation of new complex **5** as its hexafluorophosphate salt.

**Figure 3:**
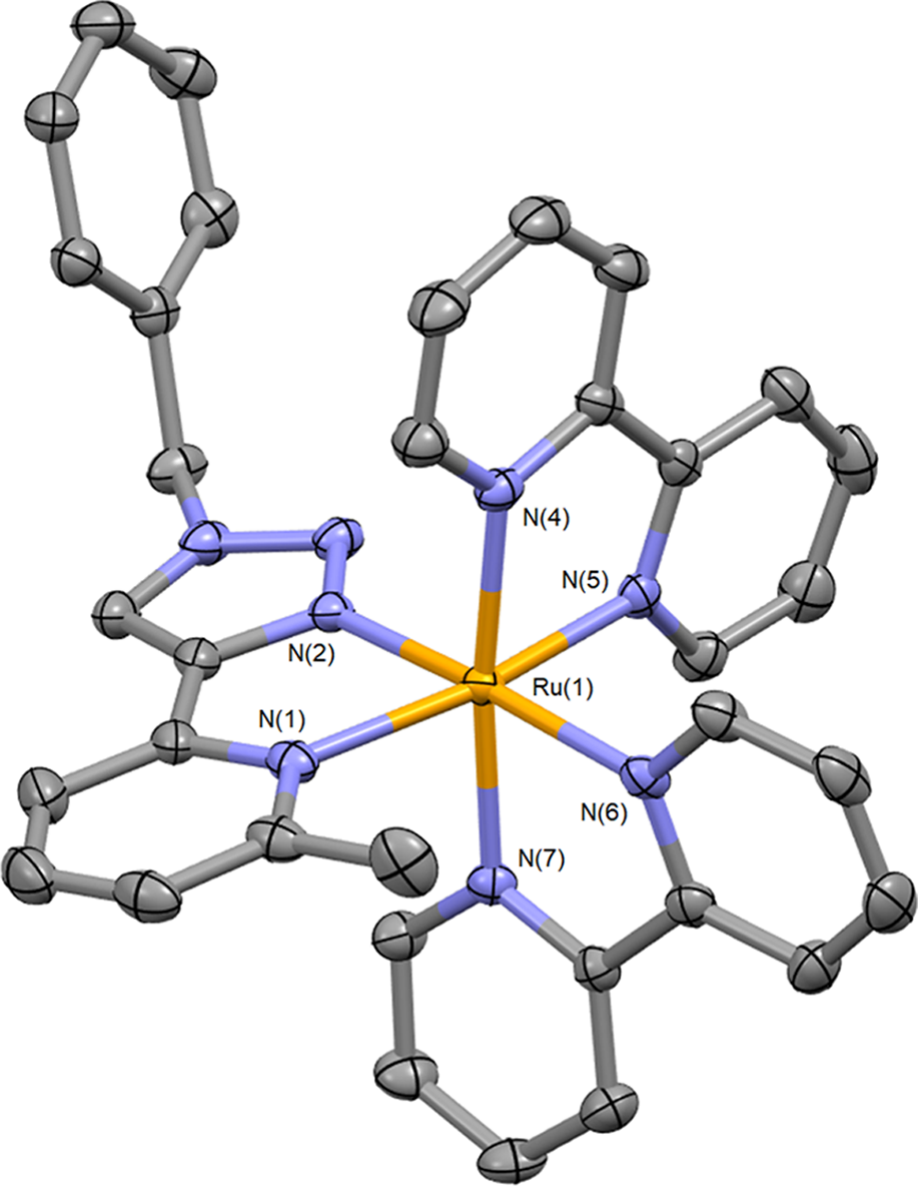
Structure of the cation for [Ru(bpy)_2_(mpytz)](PF_6_)_2_ (**5**, counterions, cocrystallized solvent molecules, and hydrogen atoms removed for clarity. Thermal ellipsoids at 50% probability. CCDC2162566).

Crystals of X-ray diffraction quality of **5** were grown, and the molecular structure was determined (Figure 3 with selected bond lengths and angles in Table 1). The Ru–N bond lengths are fairly typical for a ruthenium tris-diimine complex with values between 2.035 and 2.073 Å for the two bpy ligands. These distances are similar to those for **4**, as reported by Crowley and co-workers.^65^ The Ru–N bonds to the mpytz ligand are elongated with respect to those reported for **4** (2.16 Å for the Ru–N bond to the methylpyridine donor compared to 2.08 Å for **4**), demonstrating the steric demands imparted by the methyl group. This also results in a distortion in the neighboring bpy ligand in **5** with an intercyclic torsion angle N(6)–C–C–N(7) of 9.74°. This compares to the much smaller values of 4.15 and 2.35° for the two crystallographically unique cations in the reported structure for **4**.

**Table 1:** Selected Crystallographic Bond Lengths (Å) and Angles (deg) for [Ru(bpy)_2_(mpytz)](PF_6_)_2_

Ru(1)–N(1)	2.159(3)	N(1)–Ru(1)–N(2)	77.79(10)
Ru(1)–N(2)	2.027(2)	N(4)–Ru(1)–N(5)	79.00(10)
Ru(1)–N(4)	2.065(2)	N(6)–Ru(1)–N(7)	78.54(9)
Ru(1)–N(5)	2.034(3)	N(1)–Ru(1)–N(5)	169.89(9)
Ru(1)–N(6)	2.073(2)	N(2)–Ru(1)–N(6)	172.82(9)
Ru(1)–N(7)	2.058(2)	N(4)–Ru(1)–N(7)	173.22(9)

### Electrochemical and Photophysical Properties

**Figure 4:**
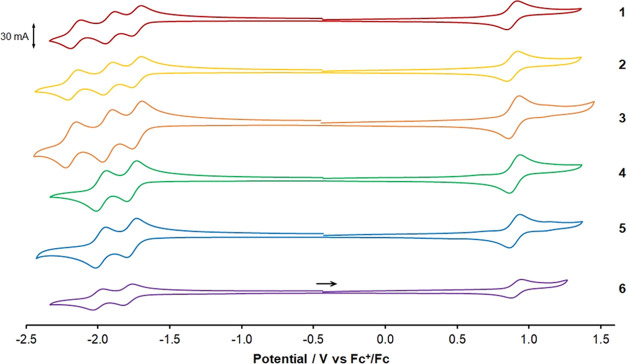
Cyclic voltammograms recorded for 1.5 mmol dm^–3^ acetonitrile solutions of **1** to **6** at 100 mV s^–1^. Potentials are shown against the Fc^+^/Fc couple (*E*_1/2_ = +0.39 V vs saturated calomel electrode (SCE)). The arrow indicates initial scan direction for all complexes.

Complexes **1** to **6** were investigated using cyclic voltammetry (Figure 4) and oxidation, and reduction potentials are provided in Table 2. Cyclic voltammograms (Figure 4) show that all processes are reversible or quasi-reversible on the basis of *I*_a_/*I*_c_ and *E*_a_–*E*_c_ values. Across the series, very little variation is observed in the potential of the Ru(II)/Ru(III) couple at approximately +0.9 V vs Fc/Fc^+^. For each of the tris(bpy)-based complexes **1** to **3**, a total of three reduction processes are observed assigned to one-electron reduction processes for each of the three bpy-based ligands. The potentials of these processes are almost invariant for **1** to **3**, indicating that methylation of one of the bpy ligands has little or no effect on the frontier orbital energies of the complexes. For the triazole-containing complexes **4** to **6**, two reduction processes are observed, assigned to reduction of the two bpy ligands in each complex. The potentials for these reduction processes are slightly shifted to more negative potentials compared to the first and second reduction processes of complexes **1** to **3**, indicating destablization of the lowest unoccupied molecular orbital (LUMO), in agreement with previous results.^58^

**Table 2:** Summarized Electrochemical Data for Complexes **1** to **6**^a^

complex	*E*_ox_/V	*E*_red_/V
**1**	+0.89	–1.73, −1.89, −2.15
**2**	+0.90	–1.71, −1.92, −2.16
**3**	+0.91	–1.71, −1.92, −2.18
**4**	+0.93	–1.74, −1.94
**5**	+0.88	–1.79, −2.01
**6**	+0.91	–1.80, −2.04

a All potentials are referenced against the ferrocene/ferrocenium couple in acetonitrile in the presence of ^*n*^Bu_4_NPF_6_ as an electrolyte.

**Figure 5:**
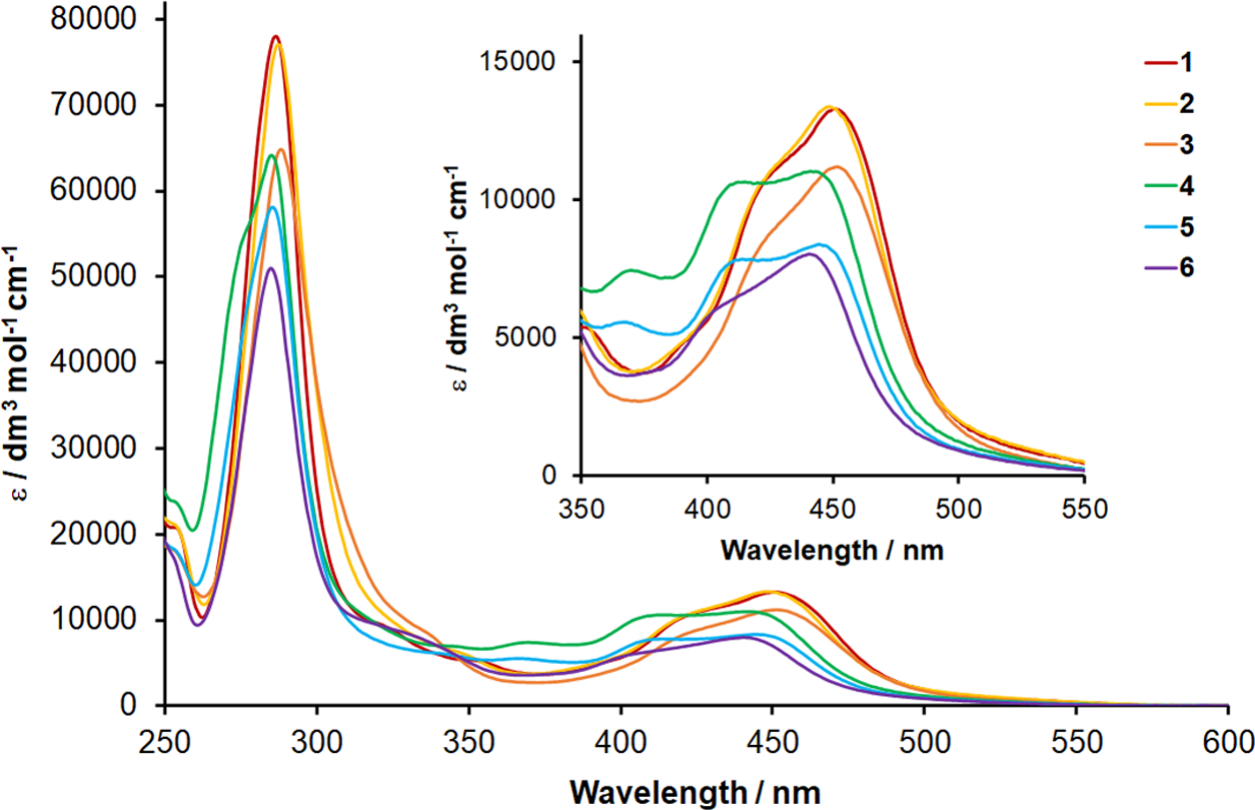
UV–visible electronic absorption spectra recorded for acetonitrile solutions of **1** to **6**. The inset shows details of charge-transfer absorption bands between 350 and 550 nm.

UV–visible absorption spectra in acetonitrile solutions were recorded for each complex (Figure 5), and summarized photophysical data are presented in Table 3. All complexes display sharp and intense bands in the region around 290 nm due to bpy-based π → π* ligand-centered transitions, with further weaker bands at lower energy assigned to singlet metal-to-ligand charge-transfer (^1^MLCT) transitions. In agreement with the electrochemical data, complexes **1** to **3** display nearly coincident ^1^MLCT absorption bands with maxima at ∼448–451 nm. Consistent with the more cathodic reduction for complexes **4** to **6** compared to that of **1**, the ^1^MLCT band is observed to blue-shift by approximately 10 nm (∼505 cm^–1^). Complexes **4** and **5** exhibit increased absorbance around 370 nm compared to **1**, assigned to ^1^MLCT transitions involving the pyridyltriazole-based ligand. For **6**, these absorptions are absent, while a band for the ^1^MLCT-based transition involving the btz ligand is discernible as a shoulder at approximately 300 nm on the low-energy side of the intense bpy-based ligand-centered band.^55,58^

**Table 3:** Summarized Photophysical Data for Complexes **1** to **6**^a^

complex	λ^abs^/nm (ε/mol^–1^ dm^3^ cm^–1^)	λ^em^/nm (77 K)
**1**	288 (78,030)	580
	450 (13,290)	628
**2**	288 (77,030)	580
	448 (13,370)	630
**3**	289 (64,770)	583
	451 (11,170)	633
**4**	285 (64,120)	569
	370 (7450)	616
	443 (11,020)	
**5**	286 (58,050)	572
	368 (5570)	620
	444 (8390)	
**6**	285 (51,030)	564
	333 (8090)	609
	440 (8010)	

a UV–visible absorption spectra were recorded in acetonitrile at room temperature, while emission data were collected at 77 K in 4:1 EtOH/MeOH glass matrices.

**Figure 6:**
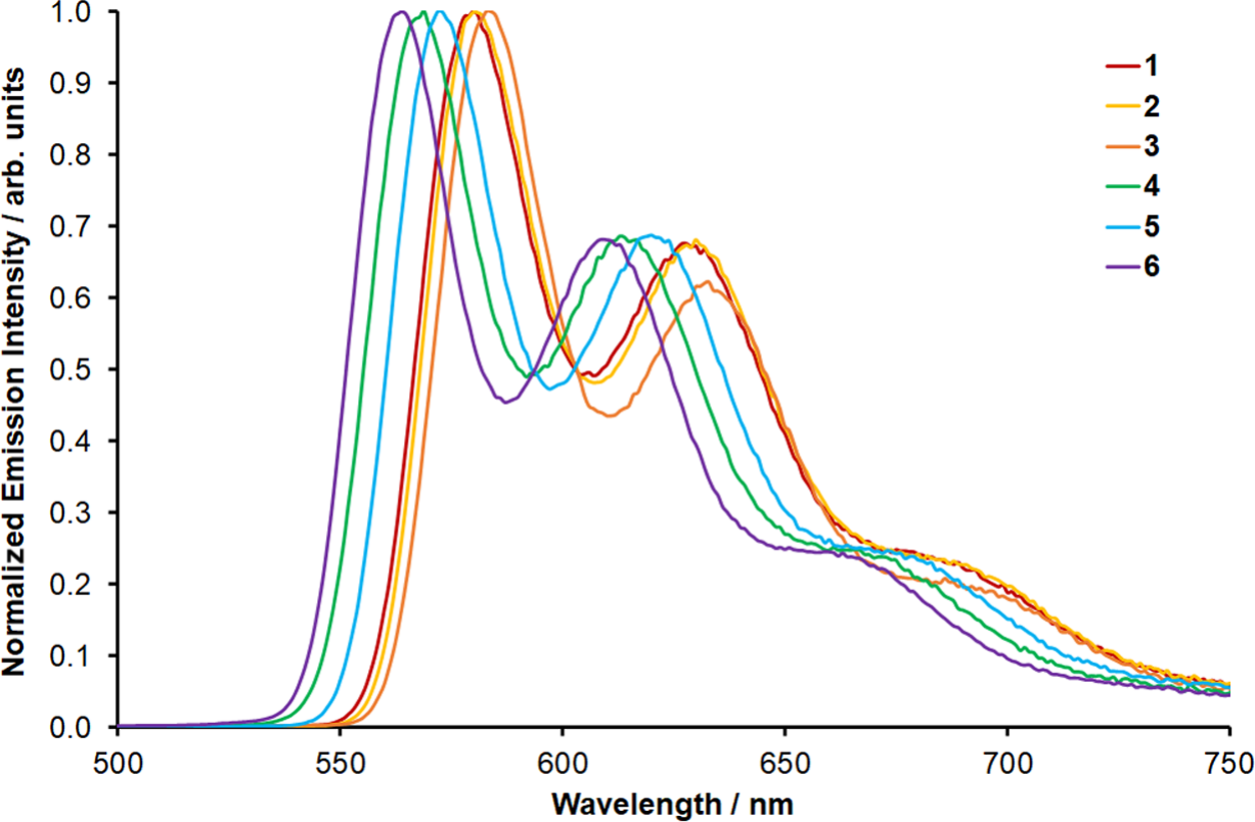
Normalized photoluminescence spectra recorded for **1** to **6** at 77 K in a 4:1 EtOH/MeOH glass.

To probe ligand effects on the ^3^MLCT state energy, luminescence spectra were recorded (Figure 6). Since the complexes, with the exception of **1**, are very weakly- or nonemissive in room temperature fluid solutions,^58,64−66^ spectra were recorded at 77 K in EtOH/MeOH glass matrices to enable direct comparison across the series. The spectra obtained for all complexes are structured, featuring clear vibronic progressions. **1** and **2** exhibit near-identically positioned emission bands (λ_max_^em^ 580 and 630 nm), with a very slight red shift observed for the emission maxima of **3**. In agreement with the electrochemical and electronic absorption data, the emission maxima of **4** to **6** are all blue-shifted relative to those of **1**, with **6** appearing at the highest energy (λ_max_^em^ 564 and 609 nm), in line with the complex having the most cathodic reduction potential. A small red shift is observed for the progressions in the spectrum for the methyl-substituted complex **5** compared to **4**.

### Transient Absorption Spectroscopy

**Figure 7:**
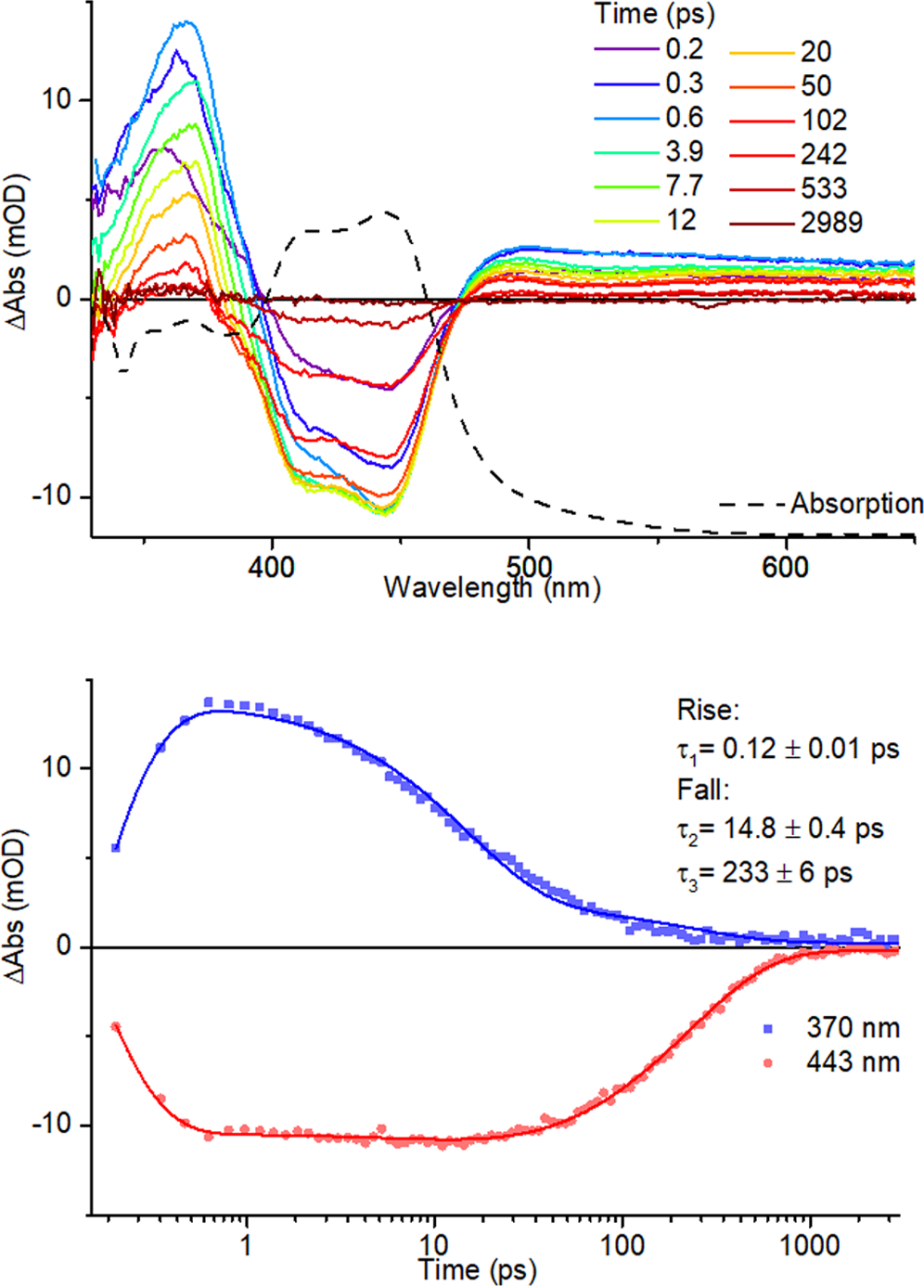
ps-Transient absorption spectra of **5** in acetonitrile solution overlaid with ground-state steady-state absorption spectrum (dashed line) (top) and kinetic traces at 370 and 443 nm (below).

Transient absorption experiments were carried out to probe the ^3^MLCT lifetimes for **1**–**6** in acetonitrile solutions at room temperature. Spectra for **1** to **4** and **6** are provided in Figures S7–S11, while those for **5** are depicted in Figure 7. Kinetic analysis was used to extract rise and decay lifetimes, which are provided in Table 4. Upon excitation, all complexes display ground-state bleach features between 400 and 500 nm, which are coincident with the ^1^MLCT absorption bands. Intense excited-state absorption (ESA) bands are also observed between 350 and 400 nm, along with a broad and less intense ESA feature beyond 500 nm, which are assigned to the ^3^MLCT state.^67^ For all complexes, a very short rise time of <0.25 ps is observed, accompanied by a second, slower process (<30 ps) manifested as a secondary rise component for **1**, **2**, and **4** and as an initial decay for **3**, **5**, and **6**, which are tentatively assigned to vibrational cooling, internal conversion, solvent reorganization, and energy redistribution processes.^67−69^ The archetypical complex **1** displays the longest lifetime, with both excited-state transient and ground-state bleach bands still evident at the end of the 3 ns time window of the experiment, as expected, given its ns-μs ^3^MLCT lifetime. The inclusion of a single methyl group in **2** results in a significant shortening of the ^3^MLCT state lifetime (τ_3_ = 2.5 ns, in agreement with previously reported data, indicating a lifetime of <10 ns^64,70^) with the replacement of one pyridine ring with a triazole donor in **4** resulting in a comparably shortened ^3^MLCT state lifetime (τ_3_ = 7.3 ns; this value agrees with data reported by Crowley and co-workers who determined that the lifetime must be <10 ns^65^). Incorporation of the second methyl group in **3** results in a 10-fold reduction in the ^3^MLCT lifetime compared to **2**. For **6**, the replacement of the pytz ligand present in **4** with the btz ligand results in a greater than 10-fold further reduction in the ^3^MLCT state lifetime (from 7.3 ns for **4** to 443 ps for **6**).

**Table 4:** Time Constants for Evolution of Transient Absorption Spectra for Complexes **1** to **6**.^a^

complex	τ_1_ (rise)	τ_2_	τ_3_
**1**	0.19 ± 0.02 ps	9.7 ± 1.3 ps	**≫3 ns**
**2**	0.13 ± 0.01 ps	27.9 ± 2.5 ps	**2.5** ± **0.7 ns**
**3**	0.16 ± 0.01 ps	7.3 ± 0.4 ps	**243** ± **14 ps**
**4**	0.24 ± 0.01 ps	15.7 ± 1.0 ps	**7.3** ± **3.2 ns**
**5**	0.12 ± 0.01 ps	**14.8** ± **0.4 ps**	233 ± 6 ps
**6**	0.21 ± 0.02 ps	18.4 ± 6.2 ps	**443** ± **17 ps**

a Time constants assigned to decay of ^3^MLCT states are highlighted in bold.

The shortest ^3^MLCT state lifetime is exhibited by **5**, incorporating both a sterically encumbering methyl substituent and a triazole donor. Interestingly, while for other complexes, hand-in-hand recovery of bleached bands occurs with decay of transient absorption features, the ground-state bleach for **5** displays a delayed recovery, following prompt evolution of excited-state transient bands. As can be seen in Figure 7, the excited-state absorption band at 370 nm has almost entirely decayed by 100 ps, while the ground-state bleach feature remains at approximately 80% of its original intensity. Delayed bleach recovery behavior has previously been documented by Hauser and co-workers for sterically encumbered ruthenium(II) complexes and ascribed to the rapid depopulation of the ^3^MLCT state to yield a metastable ^3^MC state which then decays to the ground state.^39^ We therefore have some confidence in assigning the shorter-lived spectral evolution observed for **5** (τ_2_ = 14.8 ps) as arising from conversion of the ^3^MLCT state to a ^3^MC state (along with vibrational cooling, energy redistribution processes, etc.), with the longer-lived process (τ_3_ = 233 ps) representing decay of the ^3^MC state to the ground state.

Collectively, the transient absorption data show that the steric effects imparted by methylation have a slightly greater deactivating influence on the ^3^MLCT state lifetime (i.e., **1** compared to **2**/**3** versus **1** compared to **4**/**6**) but that this is largely comparable to the electronic effect of replacing pyridine by triazole donors.

### Photochemical Reactivity

The photochemical ligand release reactivity of the complexes was investigated by UV–visible absorption spectroscopy in proteo-acetonitrile and also by ^1^H NMR spectroscopy in *d*_3_-acetonitrile for photoproduct identification. For NMR experiments, photolysis was conducted using irradiation with the mercury emission lines from a 23 W fluorescent light bulb, while for optical spectroscopic experiments, a blue light-emitting diode (LED) with an emission maximum at 446 nm was used. Under both these sets of conditions, the photolysis of **1** is exceedingly slow relative to the photochemistry observed for the remainder of the complexes, and so **1** is therefore considered “photoinert” by comparison (ϕ < 0.01%).

**Figure 8:**
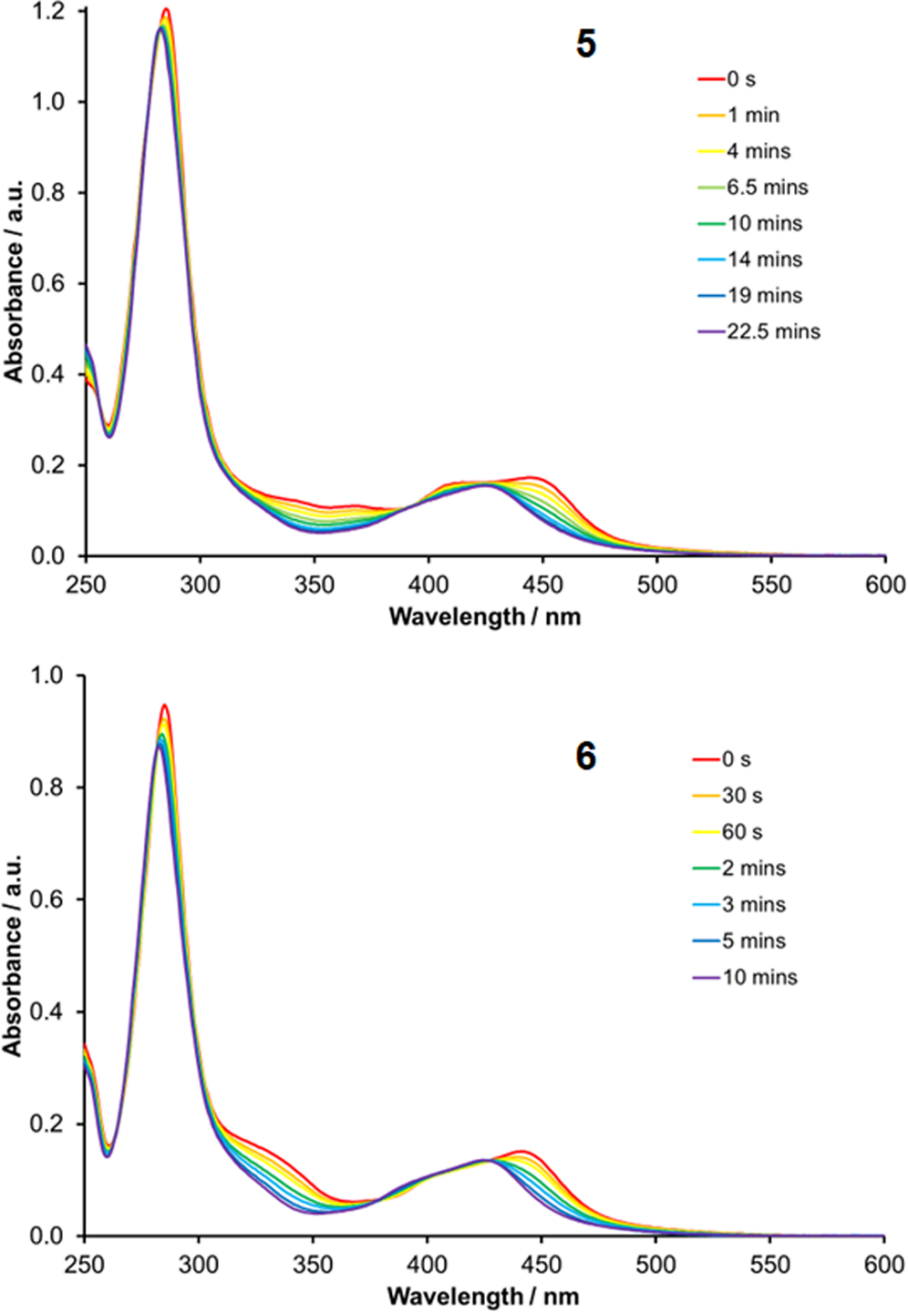
UV–visible absorption spectra of **5** and **6** recorded during photolysis in acetonitrile solution at room temperature (λ^ex^ 446 nm).

When monitored by UV–visible absorption spectroscopy, spectral features for the ^1^MLCT transitions between 400 and 500 nm for **2** to **6** are observed to evolve with clear isosbestic points, indicating a one-step photolysis process (Figures 8 and S12). For complexes **4** to **6**, this is accompanied by bleaching of the bands at 300–350 nm, consistent with loss of the triazole-containing ligand. The spectra for all complexes converge to a common band shape for the ^1^MLCT transitions, consistent with the formation of [Ru(bpy)_2_(NCMe)_2_]^2+^.^71^

The evolution of ^1^H NMR spectra is much slower than that observed by UV–visible absorption spectroscopy, consistent with the much higher concentration required, taking samples beyond the optically dilute regime. Spectral changes generally involve the loss of resonances for the starting complex and appearance of resonances for the photoproduct [Ru(bpy)_2_(NCMe)_2_]^2+^ and those of the free ligand (mbpy, dmbpy, pytz, mpytz, or btz) that has been released (Figures S13–S17 and ref (71)). In the case of **2**, photolysis is extremely slow (the sample was monitored over more than 2 weeks of continual photolysis) and appears to proceed with competitive loss of both mbpy and bpy. Further, ligand scrambling processes are evident over the prolonged photolysis, with the observed formation of **1**. However, UV–visible absorption spectra recorded during photolysis of **2** show only bleaching in the region for the ^1^MLCT absorption maximum for **2** and also **1**. Thus, due to these observations as well as the significantly prolonged time scales of photolysis, we suspect that the ligand scrambling is likely a secondary photochemical process and not indicative of what is occurring over much shorter time scales during photolysis recorded by UV–visible absorption spectroscopy on optically dilute solutions. For most of the complexes, no clear evidence for ligand-loss intermediates is observed by ^1^H NMR spectroscopy, in agreement with the isosbestic points observed in UV–visible absorption spectra. For **6**, however, weak resonances additional to those of the starting material and [Ru(bpy)_2_(NCMe)_2_]^2+^ are discernible during, but disappear on completion, of photolysis (Figure S17). This indicates the formation of a solvento intermediate of the form [Ru(bpy)_2_(κ^1^-btz)(NCMe)]^2+^ despite the observation of isosbestic points in UV–visible absorption spectra. The intermediate may exhibit very high photochemical reactivity or represent a competing minor mechanistic route, such that under the optically dilute conditions for UV–visible absorption spectroscopy, it is only formed at very low concentration and quickly consumed. At the much higher concentrations required for NMR spectroscopy, wavelengths triggering photochemical reactivity may not penetrate to the interior of the sample. Diffusional mixing between irradiated and nonirradiated regions of the sample may therefore protect an intermediate and results in a buildup to concentrations that enables its detection.

Photochemical quantum yields were determined from the evolution of UV–visible absorption spectra (Table 5) using the spectrometric approach reported by Slep and co-workers and modeled as a single-step photochemical reaction, given the observed isosbestic points.^72^ The data reveal that for **2** and **4**, the inclusion of one methyl group or replacement of one pyridine for triazole in the departing N^∧^N ligand leads to increased photochemical reactivity compared to **1**. However, the quantum yield of **4** (0.3%) is an order of magnitude larger than that for **2** (0.02%), indicating that the introduction of the triazole ring has a far greater effect in promoting photorelease despite **2** exhibiting a shorter ^3^MLCT state lifetime one presumes through ^3^MC state-mediated deactivation.

**Table 5:** Quantum Yields for Photochemical Release of N^∧^N from [Ru(bpy)_2_(N^∧^N)]^2+^ in Acetonitrile

complex	ligand	Φ/%
**1**	bpy	<0.01
**2**	mbpy	0.02
**3**	dmbpy	8.2
**4**	pytz	0.3
**5**	mpytz	0.3
**6**	btz	2.0

Interestingly, the incorporation of the additional methyl group in the mpytz ligand in **5** leads to the same rather than increased quantum yield of N^∧^N release compared to **4**, as might have been expected if steric and electronic impacts on quantum yield combine in an additive fashion. Given that **5** displays the shortest ^3^MLCT lifetime in the series as determined by transient absorption spectroscopy, efficient depopulation of the ^3^MLCT state indeed occurs but not to a photoproductive ^3^MC state. This piece of information will prove particularly important in the upcoming discussion and in the global mechanistic interpretation proposed in this work. Inclusion of sterically encumbering methyl groups on both donor rings for the departing dmbpy ligand in **3**, or incorporation of the btz ligand in **6** both lead to a further significant increase in photochemical reactivity, with quantum yields of 8.2% and 2.0%, respectively.

The apparent contradictions between transient absorption data and determined photochemical quantum yields mean that interpretation of this data is not straightforward. However, deeper insight may be offered through computational calculations on the available excited-state local minima for each complex.

### Computational Calculations

To gain deeper insight into the photophysical and photochemical properties of **1** to **6**, we carried out density functional theory (DFT) calculations on the ground and excited states of the complexes (benzyl substituents on triazole rings being modeled as methyl groups to reduce computational expense and as they will have minimal impact on photophysical properties^54^). In all cases, the highest occupied molecular orbital (HOMO) has predominantly ruthenium d-orbital character, while the LUMO has primarily bpy π* character (Figure S19). Geometries are available in the Supporting Information, and Ru–N bonds for all geometries are summarized in Table S1. For **1**, the Ru–N bonds are 2.06–2.07 Å.^73^ For **2**, five of the Ru–N bond distances are very similar to those of **1;** however, the Ru–N bond to the methyl-substituted pyridine is significantly elongated at 2.15 Å. Similarly, the Ru–N bond length to the sterically encumbered pyridine ring of the mpytz ligand in **5** (2.17 Å) is significantly elongated compared to the equivalent Ru–N bond for **4** (2.11 Å). The Ru–N distance to the dmbpy ligand in **3** (2.13 Å) is 0.05–0.06 Å longer than the Ru–N bond for the unsubstituted bpy ligands. These ground-state distortions therefore demonstrate the steric demands imparted by the methyl substituents. In comparison, the Ru–N distances for the unstrained complexes **4** and **6** have much-reduced deviation compared to **1**. Since **2** and **5** show only very little or no increase in photochemical quantum yield compared to those of **1** and **4**, respectively, these ground-state geometry elongations offer little insight into the relationships governing the efficiency of the observed photochemistry. Excited-state optimizations are therefore required to derive additional arguments for the rationalization of the varying behaviors observed.

**Figure 9:**
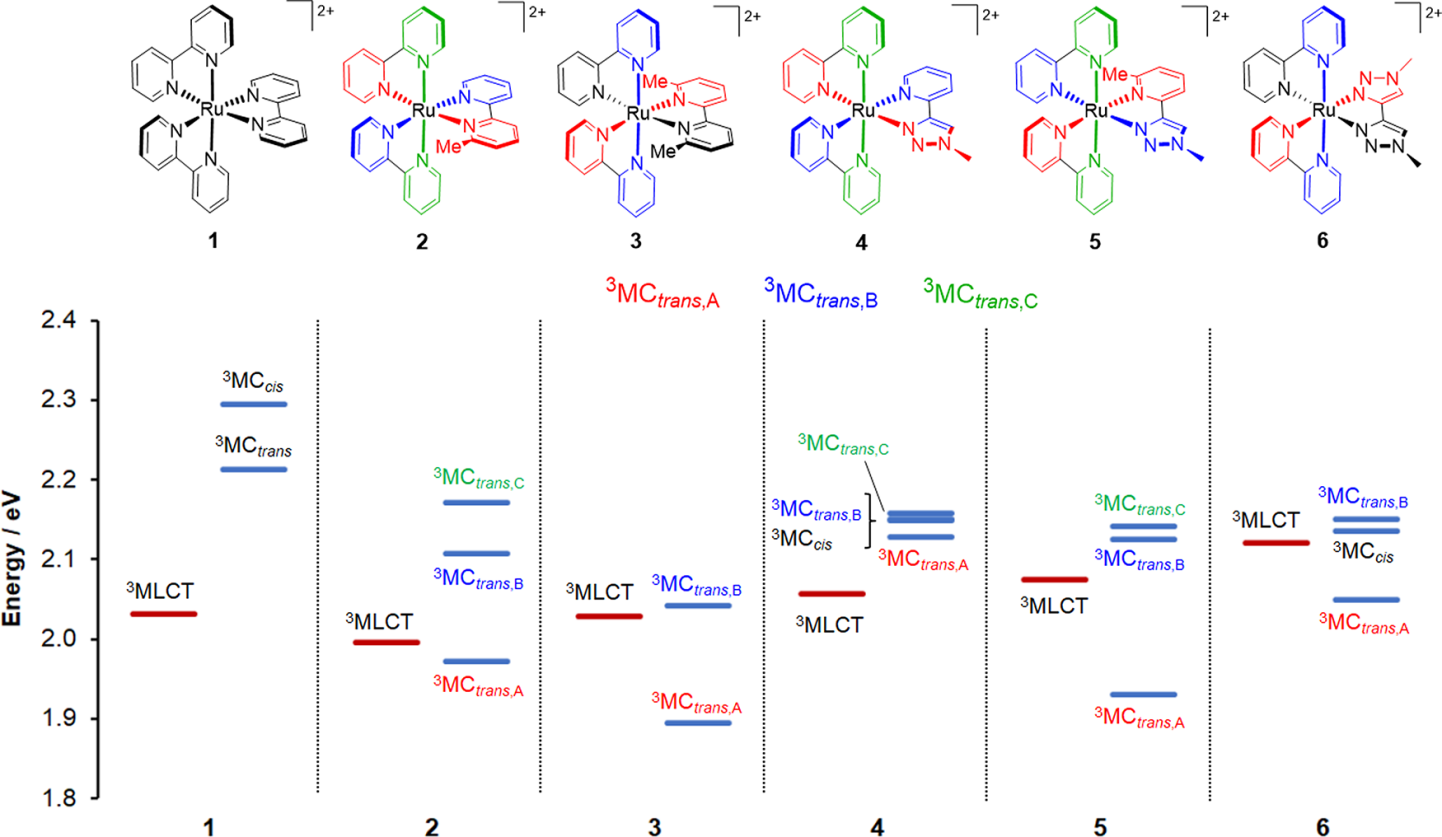
Definition of the inequivalent ^3^MC_trans,A_, ^3^MC_trans,B_, and ^3^MC_trans,C_ states for **2** to **5** based on color coding of pyridine donors and corresponding Ru–N bonds along which respective axial elongation distortions occur (top). Relative energies of optimized ^3^MLCT and ^3^MC states for **1** to **6** quoted relative to their respective optimized ground states (*E* = 0.0 eV) (bottom). For the ^3^MC_cis_ states for **4** and **6**, the ligand that is repelled is the pytz and btz ligand, respectively.

The ^3^MLCT state geometries for each complex were optimized, as well as geometries for possible ^3^MC states, using initial guess geometries based on structural parameters from our previous studies.^59,60^ The energies of the optimized ^3^MLCT and ^3^MC states of **1** to **6**, relative to the energies of their respective ground states, are depicted in Figure 9. The ^3^MLCT states for all complexes exhibit one singly occupied natural orbital (SONO) of ruthenium d-orbital character and a second SONO (SONO + 1) of bpy π* character (Figure S20). Mulliken spin densities of ∼0.99 on the ruthenium atom confirm the charge-transfer nature of these ^3^MLCT states. In agreement with expectations based on electrochemical and photophysical data, the ^3^MLCT states of the pyridyltriazole-based complexes **4** and **5** are higher in energy than that of **1** and the methylated bpy containing complexes **2** and **3**, with the most destabilized ^3^MLCT state arising for **6**.

**Figure 10:**
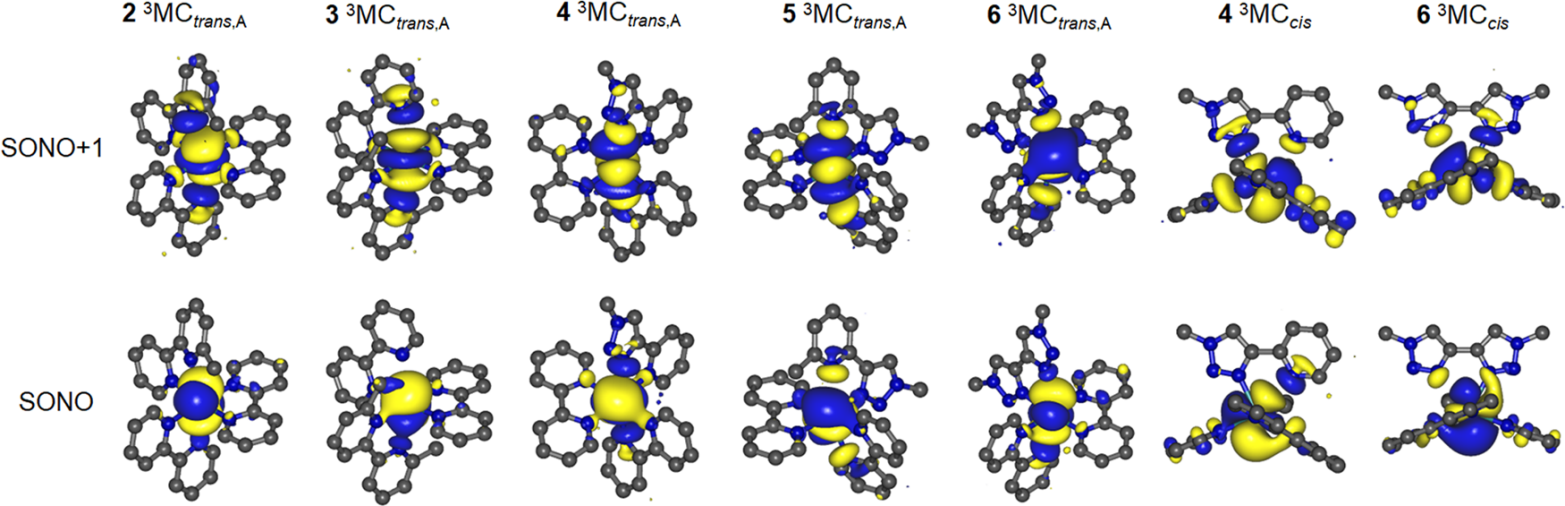
Singly occupied natural orbitals (SONOs) for the lowest energy ^3^MC states of **2** to **6** (^3^MC_trans,A_) and those for the ^3^MC_cis_ states of **4** and **6**.

**Figure 11:**
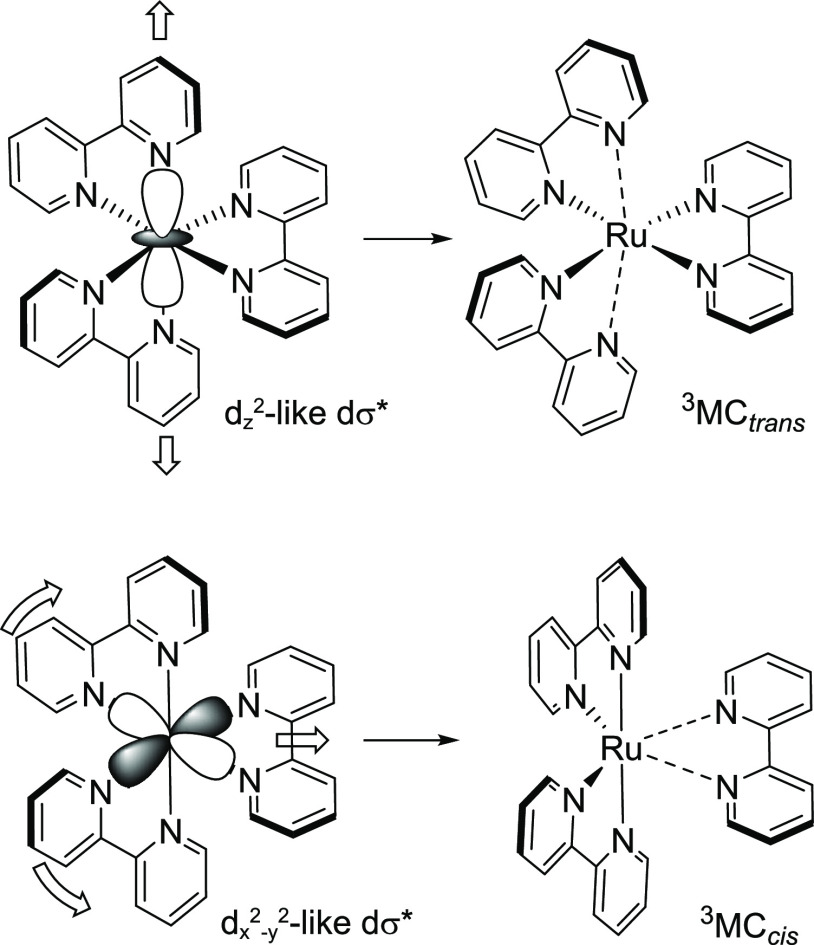
Classification of hexacoordinate ^3^MC states as ^3^MC_trans_ and ^3^MC_cis_ based on the population of either d*_z_*_^2^_-like or d*_x_*_^2^–*y*^2^_-like dσ* orbitals, respectively.

For ^3^MC states, relevant SONOs are plotted in Figures 10 and S21, and structural parameters are collated in Table S1. For Ru(II) tris-bidentate complexes, we have previously classified hexacoordinate ^3^MC states into two principal types. First, axial elongation to two Ru–N bonds situated trans to one another are characterized by population of a d*_z_*_^2^_-like dσ* orbital^16^ and are thus termed ^3^MC_trans_ states (Figure 11).^60^ Second, population of a d*_x_*_^2^–_*_y_*_^2^_-like dσ* orbital may result in elongation of two Ru–N bonds situated cis to one another, which is accompanied by a widening of the N–Ru–N angle for the Ru–N bonds trans to those elongated (Figure 11). These states we term ^3^MC_cis_ and have previously identified ^3^MC_cis_ states for **1** and **6**.^59,60^

As the three N–Ru–N axes of **1** are equivalent, the three possible axial elongations give rise to equivalent ^3^MC_trans_ states. However, for the heteroleptic complexes **3** and **6** that incorporate a symmetrical dmbpy or btz ligand, respectively, there are two unique types of N–Ru–N axis and thus two unique types of ^3^MC_trans_ state, which were optimized in each case (elongation for N(bpy)–Ru–N(dmbpy/btz) and N(bpy)–Ru–N(bpy) axes, termed ^3^MC_trans,A_ and ^3^MC_trans,B_, respectively, and as defined in Figure 9). For **2**, **4**, and **5**, which incorporate an asymmetric third ligand, the three N–Ru–N axes are unique, and thus, three distinct ^3^MC_trans_ states are expected (^3^MC_trans,A_, ^3^MC_trans,B_, and ^3^MC_trans,C_, Figure 9), which were indeed located, in each case.

As one of the bpy ligands in **1** is replaced by pytz or btz in **4** and **6**, respectively, ^3^MC_trans_ states are progressively stabilized with increasing triazole content. The ^3^MC_trans,A_ states are the lowest in energy for **4** and **6**, involving elongation of the Ru–N(triazole) bond. For **1** and **4**, all of the ^3^MC states lie higher in energy than the ^3^MLCT state, whereas for the other complexes, the ^3^MLCT state energies sit within the range of energies for, and are thus straddled by, the ^3^MC states. For each of the complexes **2**, **3**, and **5** incorporating (a) ligand methyl substituent(s), the ^3^MC_trans,A_ states involving elongation of the Ru–N bond to the methylated pyridine donor are significantly stabilized with respect to the other ^3^MC states and lie 0.03, 0.13, and 0.14 eV below their respective ^3^MLCT states.

We attempted to locate ^3^MC_cis_ states on the *T*_1_ PES for each complex in which the Ru–N bond elongations occur so as to repel the departing ligand. While the geometry for a ^3^MC_cis_ state for **4** was indeed located, such minima could not be found for **2**, **3**, and **5**. This presumably stems from the steric encumbrance imparted by the departing ligand methyl substituents that would inhibit the widening of the angle between the two bpy ligands. For both **4** and **6**, the ^3^MC_cis_ states sit within the range of energies for their respective ^3^MC_trans_ states and are highly accessible from the ^3^MLCT state.

The trend for the relative energies of the ^3^MLCT and lowest energy ^3^MC states of the complexes is in good agreement with the reduced ^3^MLCT state lifetime resulting from either increasing the steric congestion through incorporation of ligand methyl substituents, or through replacement of pyridine donors with triazole. Both act to make population of ^3^MC states from the ^3^MLCT state more favorable. However, it is noted that this does not translate to the trend in photochemical quantum yields for ligand release.

### Rationalizing ^3^MLCT Lifetimes versus Photochemical Reactivities

We have previously postulated that ^3^MC_trans_ and ^3^MC_cis_ states for [Ru(N^∧^N)_3_]^2+^ complexes, while both having the potential to deactivate ^3^MLCT states, have differing preferential roles with respect to facilitating ground-state recovery versus promoting photochemical reactivity.^51^ While these states exhibit elongated Ru–N bonds, they nevertheless remain hexacoordinate, and in ^3^MC_trans_ states, the metal center remains significantly shielded from a potential incoming ligand. We have suggested that ^3^MC_trans_ states are therefore more prone to facilitating ground-state recovery than in going on to form photoproducts.^51,60^ On the other hand, ^3^MC_cis_ states, in which both Ru–N bonds for the departing ligand are elongated, also exhibit an open quadrant created by the widening of the angle between the “spectator” ligands, thus potentially exposing the metal center to incoming ligands. In our previous work, we have proposed that ^3^MC_cis_ states are therefore far more prone to result in photochemical reactivity.^51,60^ Indeed, a ^3^MC_cis_ state was shown to be crucial in mediating the observed formation of the ligand-loss intermediate and final photoproducts *trans*-[Ru(bpy)(κ^2^-btz)(κ^1^-btz)(NCMe)]^2+^ and *trans*-[Ru(bpy)(btz)(NCMe)_2_]^2+^, respectively, in acetonitrile solution, in which the retained bidentate bpy and btz ligands are coplanar.^36,37^

For **1**, the fact that the lowest ^3^MLCT state lies significantly below the energies of the ^3^MC states agrees with the comparatively low photochemical reactivity of the complex. Incorporation of a single methyl group on one bpy ligand in **2** leads to a significant elongation in the ground-state Ru–N bond for the pyridine ring bearing the methyl group. Thus, one would expect this steric encumbrance to result in a lowering in energy of ^3^MC_trans,A_, whose elongation is aligned with this axis. Indeed, calculations reveal a stabilization of the ^3^MC states for **2** compared to those of **1**, particularly so for the ^3^MC_trans,A_ state, to the point that it is now lower in energy than the ^3^MLCT state. As is evident from the transient absorption data, the stabilization of ^3^MC_trans,A_ for **2** results in rapid deactivation of the ^3^MLCT state (τ_3_ = 2.5 ns). The still very low photochemical quantum yield for release of the mbpy ligand from **2** is, however, in agreement with ^3^MC_trans,A_ being prone to facilitating ground-state recovery over photochemical reactivity.

For **4**, the replacement of one pyridine for a triazole donor in the pytz ligand leads to a slight destabilization of the ^3^MLCT state and a stabilization of the ^3^MC states compared to **1**. Thus, the closer proximity of the ^3^MLCT state to the closely spaced set of ^3^MC states (including ^3^MC_cis_) is in agreement with the observed shortened lifetime of **4** (τ_3_ = 7.3 ns) compared to **1**. With the ^3^MC states lying just above the ^3^MLCT state, this is also in agreement with a longer ^3^MLCT state lifetime compared to **2**. However, despite the reduced accessibility of the ^3^MC states for **4** compared to **2**, it is noted that **4**, for which a ^3^MC_cis_ state local minimum could be located, shows a 10-fold higher photochemical quantum yield for N^∧^N loss.

For **5**, the inclusion of a methyl group in the mpytz ligand leads to the shortest ^3^MLCT lifetime for all complexes in the series (τ_2_ = 15 ps), significantly shorter than that of **4**. In this case, the long-lived decay process instead stems from the return of a metastable ^3^MC state to the ground state (τ_3_ = 233 ps), as evidenced from the prompt decay of transient absorption bands for the ^3^MLCT state and delayed ground-state bleach recovery. In agreement with **2**, calculations reveal the lowest energy ^3^MC state to involve elongation along the axis containing the methyl-substituted pyridine donor (^3^MC_trans,A_). However, the increased rate of ^3^MLCT deactivation compared to **4**, with the apparent promotion of the population of ^3^MC_trans,A_, does not translate into increased photochemical reactivity, again highlighting its preferential role in promoting ground-state recovery.

For the btz-containing complex **6**, the inclusion of a second triazole ring leads, again, to a shortening of the ^3^MLCT state lifetime (τ_3_ = 443 ps) compared to **4**. Calculations reveal that the ^3^MC_trans,A_ state is slightly lower in energy than the ^3^MLCT state (which is itself further destabilized relative to that of **4**), which is almost isoenergetic with the ^3^MC_cis_ state. Thus, the ^3^MLCT state is rapidly depopulated, and a further 10-fold enhancement of photochemical quantum yield for ligand release is observed, but in the absence of any steric encumbrance.

The increased photochemical reactivity of **4** and **6** relative to **1** would therefore seem to correlate with the existence and accessibility on the *T*_1_ PES of ^3^MC_cis_ states. These states could not be located for **2** and **5**, seemingly accounting for their limited photoreactivity. As discussed above, ^3^MC_cis_ states are important for the formation of trans photoproducts for [Ru(bpy)(btz)_2_]^2+^. As the angle between the retained bpy and btz ligand widens, the lack of steric impediment for the retained btz ligand enables it to become fully coplanar with the bpy ligand. On the other hand, while *trans*-[Ru(bpy)_2_(L)_2_]^2+/0^ complexes are known,^74,75^ coplanarization of two bpy ligands is nonetheless inhibited by steric interactions between the H6 and H6′ protons of the two bpy ligands and leads to severe distortions. Hence, *cis*-[Ru(bpy)_2_]-containing photoproducts predominate. However, as we outline below and have shown previously, ^3^MC_cis_ states may contribute to the formation of both cis and trans photoproduct formation.^76^

As the reader may note, we have yet to discuss complex **3** and will do so during the next section.

### Broader Mechanistic Implications

Early work, for example, by Van Houten^27,77^ and Meyer,^23,78,79^ provided compelling evidence for the photochemistry of [Ru(bpy)_3_]^2+^ and related complexes as proceeding via a dissociative or interchange-dissociative mechanism. For the ligand substitution of the thiocyanate salt, evidence by UV–visible absorption spectroscopy is observed for an unstable intermediate, presumably of the form [Ru(bpy)_2_(κ^1^-bpy)(NCS)]^+^.^24^ However, intermediates are not observed under other conditions with different incoming ligands. Meyer noted the dependence on the nature of the incoming ligand, attributing this to competition between coordination of the incoming ligand to the pentacoordinate [Ru(bpy)_2_(κ^1^-bpy)]^2+^ with favorable rechelation of the κ^1^-bpy ligand. On the other hand, Glazer and co-workers reported evidence of associative photochemical ligand substitution for the complex [Ru(bpy)_2_(dmdppz)]^2+^ (dmdppz = 3,6-dimethyldipyridylphenazine).^80^ This was attributed to the coordination of a solvent ligand to the electron-deficient Ru(III) center of the ^3^MLCT state. However, in the same work, the photochemical behavior of **3** suggested dissociative character. In work on chelating bisthioether complexes of the form [Ru(bpy)_2_(S^∧^S)]^2+^, Turro showed that the ^3^MLCT states can show significant dissociative character, with large elongations of the Ru–S bonds.^81^ This and other prior work from a number of groups have therefore shown that the rate and efficiency of photochemical ligand substitution in Ru(II) trischelate complexes can show a dependence on the nature of the departing ligand, the incoming ligand, solvent, and temperature. Further, this demonstrates that photochemical ligand substitution in these systems may operate by a variety of mechanisms.

**Figure 12:**
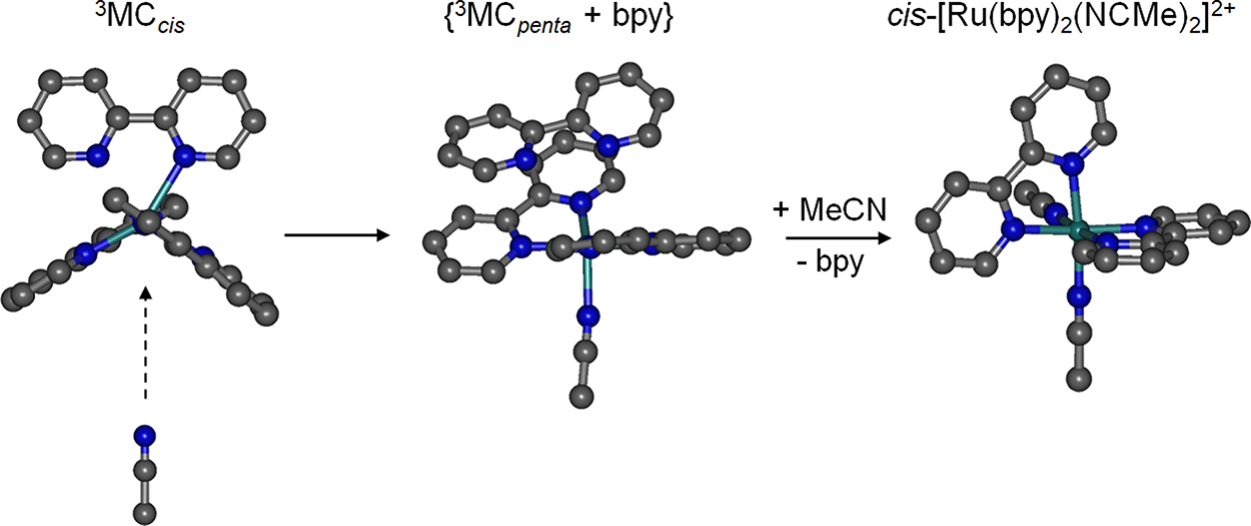
Concomitant solvent coordination and formal bpy release for **1** via a pentacoordinate ^3^MC state van der Waals adduct. Geometries of the cations depicted are DFT-optimized minima from ref (76).

We have recently reported computational studies on the full photosolvolysis mechanism for **1**, including routes involving and circumventing κ^1^-bpy solvento intermediates, as a model system in acetonitrile from which we may draw parallels with other more photoreactive complexes.^76^ These studies provided three important insights. (1) The ^3^MC_cis_ state was shown to be able to contribute to product formation pathways for both *cis* and *trans*-[Ru(bpy)_2_(NCMe)_2_]^2+^. (2) Minimum energy path optimizations revealed that there are potential low-energy pathways to direct solvent capture by ^3^MC states without contravention of Wigner’s rules.^82^ Electrostatic repulsion between the lone pair of an approaching solvent molecule and the unpaired electron located in the dσ* orbital can result in a switching to a triplet state of alternative character (e.g., another ^3^MC state with elongation of other Ru–N bonds or a ^3^MLCT state), which enables solvent coordination. (3) In the triplet state solvent molecule capture for ^3^MC_cis_, the solvent can approach the Ru center in the open quadrant between the two “spectator” ligands and on the opposing side of the metal center to the departing bpy ligand whose Ru–N bonds are both elongated. During this process, rather than form an approximately, though distorted, coplanar arrangement, the “spectator” bpy ligands rearrange their orientation, while the Ru–N bonds to the departing bpy ligand formally rupture to yield a pentacoordinate ^3^MC state of form ^3^[Ru(bpy)_2_(NCMe)]^2+^ in which the departing bpy ligand remains associated in a van der Waals adduct ({^3^MC_penta_ + bpy} in Figure 12). Due to steric interactions between the two retained bpy ligands that prevent true coplanarity, one of the bpy ligands slides up over the other in forming this pentacoordinate ^3^MC state, which then favors the formation of the *cis*-[Ru(bpy)_2_(NCMe)_2_]^2+^ photoproduct.

The striking observation from these insights is that both solvent capture and rupture of both Ru–N bonds with formal release of departing ligand may occur as part of a single concerted photochemical process without the need for formation of the κ^1^-N^∧^N ligand-loss primary photoproduct. This would agree with the lack of intermediates observed for many complexes and the isosbestic behavior, when monitored by UV–visible absorption spectroscopy, so this pathway may dominate in many cases.

**Scheme 2:**
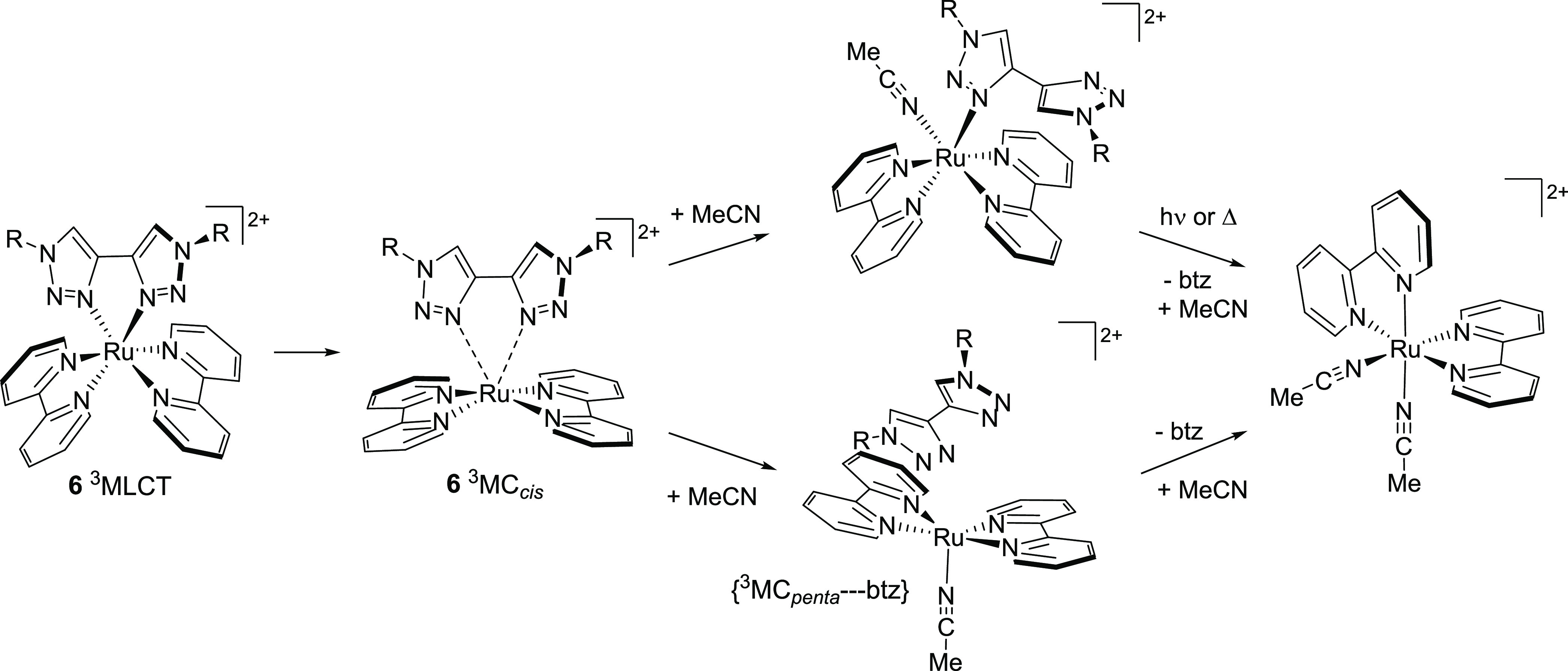
Proposed Excited-State Mechanisms for Step-Wise and Concomitant Solvent Coordination and Departing Ligand Release Illustrated for **6** A cis arrangement of the acetonitrile and κ^1^-btz is depicted in the upper pathway on the basis that a trans arrangement would generate significant steric strain;^76^ however, a trans isomer could also be envisaged.

On the other hand, a step-wise photochemical ligand release indeed proceeds with observation of a κ^1^-N^∧^N solvento intermediate in several other cases (Scheme 1).^83^ For example, for [Ru(bpy)_2_(S^∧^S)]^2+^-type complexes where S^∧^S is a bisthioether ligand with a flexible linker^84^ and for [Ru(bpy)_2_(3,3′-dimethyl-2,2′-bipyridyl)]^2+^ where a steric clash between the methyl substituents results in a “spring-loaded” photodechelation, which favors solvento intermediate formation.^32^ Weak resonances for an intermediate are observed for **6**, though isosbestic behavior when monitored by UV–visible absorption spectroscopy suggests that this species never builds up to any appreciable concentration. While one or other of these mechanistic pathways may dominate for a particular complex under a given set of conditions (solvent, identity of the incoming ligand, etc.), it is possible that both may operate competitively for some systems. These possible pathways are illustrated in Scheme 2 for **6**.

We have yet to discuss complex **3**, which of course, displays the highest photochemical reactivity in the series. Bearing two methyl substituents in the dmbpy ligand, the complex is highly strained and exhibits significant distortion in the ground state.^66^ For **2** and **5** bearing a single methyl substituent, population of the ^3^MC_trans,A_ state relieves the steric strain present in the ground and ^3^MLCT states as the Ru–N bond to the methyl-substituted pyridine ring elongates. For **3**, however, there will still be a strain in the ^3^MC_trans,A_ state due to the second methyl substituent on the fully coordinated pyridine ring of the dmbpy ligand. As alluded to above, the formation of a ^3^MC_cis_ state will also be inhibited, with the methyl groups of dmbpy precluding the required angular opening between the two spectator bpy ligands. It is therefore likely that the “brute force” approach of the two sterically encumbering methyl substituents pushes the complex over a tipping point, beyond which photochemical reactivity is shunted into operating in a different mechanistic regime. Rotation about the intercyclic C–C bond of the dmbpy ligand may enable access to photoproductive pentacoordinate trigonal bipyramidal ^3^MC states (^3^MC_penta_, which have not been calculated in this study (Scheme 2)).^85^ To underline this, Meijer has shown that the related 2,9-dimethylphenanthroline (dmphen) complex [Ru(bpy)_2_(dmphen)]^2+^, in which this bond rotational motion is not possible (and where ^3^MC_cis_ state is presumably heavily disfavored), shows substantially reduced photochemical reactivity compared to **3** (ϕ ≤ 0.5%). The complex in fact undergoes competitive photorelease of dmphen and bpy.^86,87^

**Scheme 3:**
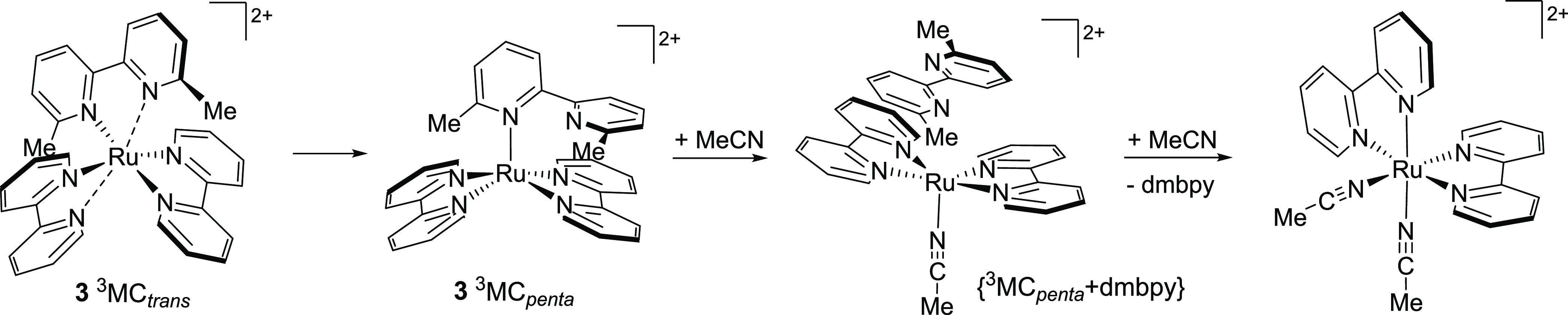
Possible Mechanism for the Concerted Solvent Addition and dmbpy Release for **3**

Formation of a pentacoordinate ^3^MC state for **3** might be expected to result in the formation of a ligand-loss intermediate photoproduct of the form [Ru(bpy)_2_(κ^1^-dmbpy)(NCMe)]^2+^; however, the occurrence of isosbestic points in UV–visible absorption spectra during photolysis and no detection of an intermediate is not consistent with this. The steric pressure imparted by the methyl substituent of the coordinated pyridine ring of the dmbpy ligand would result in the solvento intermediate being highly strained, and thus, the dmbpy may undergo rapid thermally driven dissociation soon after dechelation if a pentacoordinate intermediate is formed at all. On the other hand, this same steric strain for a resultant ^3^MLCT or hexacoordinate ^3^MC states could facilitate a concomitant solvent capture and ligand release process to yield ^3^*[Ru(bpy)_2_(NCMe)]^2+^ as a van der Waals adduct with dmbpy in a related fashion to that proposed originally for **1** (Scheme 3).^76,82^ While steric strain in **2** and **5** would be largely relieved on forming the ^3^MC_trans,A_ state, the lack of a ^3^MC_cis_ state may mean that photochemistry for these complexes may proceed by a similar ^3^MC_penta_ mechanism but with much-reduced efficiency due to reduced propensity to become pentacoordinate. Interestingly, Kayanuma has very recently reported computational studies on the photoaquation of **3**, detailing a similar ^3^MC state solvent capture mechanism. This involved a calculated pathway in which a water ligand enters cis to the κ^1^-dmbpy ligand at the ^3^MC_trans,A_ state with subsequent dissociation of dmbpy to yield a ^3^MC state of the form [^3^Ru(bpy)_2_(OH_2_)]^2+^.^88^

**Figure 13:**
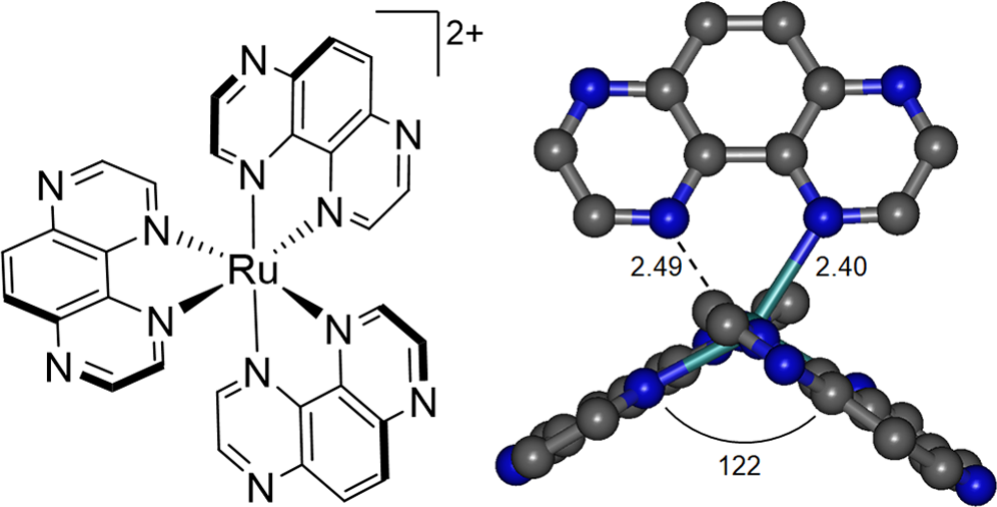
Structure of [Ru(TAP)_3_]^2+^ and the optimized geometry of its ^3^MC_cis_ state (elongated bond lengths in Å and N–Ru–N angle in deg).^89^

One also could venture that such bond rotations and pentacoordinate ^3^MC states could come into play for **4** and **6** rather than photochemistry stemming from the population of the hexacoordinate ^3^MC_cis_ state. While this is possible, we have also recently reported the photochemistry of the tetraazaphenanthrene (TAP) complex [Ru(TAP)_2_(btz)]^2+^ and also noted the known photochemical reactivity of the homoleptic complex [Ru(TAP)_3_]^2+^.^18,89,90^ [Ru(TAP)_2_(btz)]^2+^ is observed to undergo photochemical loss of TAP (competitively to the dominant loss of btz) to form *trans*-[Ru(TAP)(btz)(NCMe)_2_]^2+^ while [Ru(TAP)_3_]^2+^ releases TAP to form *cis*-[Ru(TAP)_2_(NCMe)_2_]^2+^. In both cases, DFT calculations enabled optimization of ^3^MC_cis_ states in which a TAP ligand is repelled (Figure 13). Thus, for [Ru(TAP)_3_]^2+^, the respectable quantum efficiency for TAP release (ϕ = 2%), the lack of a suitable C–C bond about which rotation may occur to favor the formation of pentacoordinate species, and the optimization of a ^3^MC_cis_ state are supportive of photochemistry occurring through this state and without the involvement of a ground-state κ^1^-TAP intermediate.

The data discussed here, in conjunction with those reported previously, provide compelling support for a novel mechanistic regime for photochemical ligand release from ruthenium(II) tris-diimine type complexes. While other competing mechanisms may also operate, photochemistry for these complexes is proposed to proceed through the population of a ^3^MC_cis_ state which undergoes concomitant solvent capture and formal release of the departing ligand in a single excited-state process that negates the need to invoke a sequential mechanism with intervening involvement of ground-state ligand-loss intermediate complexes bearing a monodentate departing N^∧^N ligand.

## Conclusions

Photochemically reactive ruthenium(II) complexes are of significant interest for application in photoactivated chemotherapy (PACT). However, the excited-state mechanistic details of photochemical ligand release from tris-bidentate ruthenium(II) complexes have been the subject of considerable ambiguity. ^3^MC states which mediate these processes are either extremely short-lived or are spectroscopically dark, which makes their direct investigation challenging. We have reported here a systematic study through combined steady-state and time-resolved spectroscopic and computational chemistry approaches to elucidate structure–property relationships, which determine the photoreactivity for ruthenium(II) tris-diimine complexes. We have probed the contribution from steric strain, as well as the electronic effect of replacement of pyridine donors for triazole, on the deactivation of the ^3^MLCT state and on the promotion of photochemical ligand release.

Introduction of a single methyl substituent adjacent to the coordinating N-atom of a bpy-based ligand results in steric strain, which dramatically stabilizes ^3^MC_trans_ states. While ^3^MC_trans_ states in these complexes promote rapid deactivation of their ^3^MLCT states, they counterintuitively do not promote significant enhancement of photochemical reactivity but instead, favor ground-state recovery.

What seems evident is that for nonstrained complexes with a small ^3^MLCT–^3^MC gap, the accessibility of ^3^MC_cis_ states leads to a far greater propensity for photochemical reactivity. A photoreactive excited-state mechanism in which solvent coordination and formal ligand release occur at the ^3^MC_cis_ state at the same time negates the need for commonly invoked κ^1^-N^∧^N ligand-loss ground-state intermediate complexes. While more rarely observed, they are nonetheless observed in some cases, as discussed above, and so one-step/no intermediate and two-step/with intermediate mechanisms may compete.

Where the complex is severely strained in dmbpy complexes, for example, where the departing ligand contains two sterically encumbering methyl substituents, the population of ^3^MC_cis_ states is inhibited, but photochemistry may proceed via alternative pathways involving coordinatively unsaturated pentacoordinate ^3^MC states. What is clear is that a thorough understanding of the structure–property relationships that determine the nature of the ^3^MC states that are accessible to a given complex is essential for the rational design of efficiently photoreactive complexes for PACT. Improved understanding of these structure–property relationships may facilitate identification of potential departing ligands for the design of high potency and efficiently photoreactive PACT complexes without incorporating steric strain and where the departing ligand itself is a pharmacologically active species.

The excited-state landscape navigated by ruthenium(II) complexes during photochemical ligand substitution is clearly complex, with the route taken and thus the mechanistic pathway that predominates dependent on the nature of the departing, as well as incoming ligands. Further, while this work provides illuminating insights into the photochemistry of Ru(II) complexes, computational studies are based on static DFT calculations. Future studies will be required to refine our understanding of these systems, which will require dynamic quantum mechanical calculations to observe how the critical ^3^MC states identified evolve.^91^ We are currently in the planning stages for such work.

## Experimental Section

### General Methods

Known complexes were prepared by literature methods.^31,58,64,65^ NMR spectra were recorded on a Bruker Ascend 400 MHz spectrometer, with all chemical shifts being reported in ppm and referenced relative to the residual solvents signal (CHCl_3_, ^1^H: δ 7.26, ^13^C δ 77.16; MeCN ^1^H: δ 1.94, ^13^C δ 1.32, 118.26). Mass spectra were recorded at high resolution on an Agilent 6210 TOF instrument with a dual ESI source or on a Bruker Q-ToF mass spectrometer. UV–visible absorption spectra were recorded on an Agilent Cary-60 spectrophotometer utilizing quartz cuvettes of 10 mm path length. Photoluminescence spectra were recorded on a Horiba Fluoromax-4 spectrophotometer at 77 K in a 4:1 EtOH/MeOH glassing mixture.

### Electrochemistry

Cyclic voltammograms were measured using a PalmSens EmStat3 potentiostat with PSTrace electrochemical software. Analyte solutions with a typical concentration of 1.5 mmol dm^–3^ were prepared using dry MeCN, freshly distilled from CaH_2_. The supporting electrolyte was N^*n*^Bu_4_PF_6_, being recrystallized from EtOH and oven-dried prior to use with a typical solution concentration of 0.2 mol dm^–3^. The working electrode was a glassy carbon disk; Pt wire was used as a counter electrode, and the reference electrode was Ag/AgCl, being chemically isolated from the analyte solution by an electrolyte-containing bridge tube tipped with a porous frit. All potentials are quoted relative to the Fc^+^/Fc couple as an internal reference.

### Ligand Release Photochemistry

Photolysis experiments were carried out by irradiating the appropriate solutions contained within either NMR tubes with a compact 23 W fluorescent light bulb (Hg) or within 10 mm pathlength quartz cuvettes with light from a blue LED (Thorlabs, LED450LW, λ = 446 nm) at a forward current of 50 mA (2.7 V) provided by a direct current power supply (RS-Components, RS-3005D). Light from the blue LED was delivered to the sample through a liquid light guide (17 ± 1 mW at the exit of the light guide). Samples were maintained at room temperature (25 °C) throughout the measurements with the aid of a Peltier temperature-controlled cuvette holder or an electronic fan (NMR samples). The determination of photochemical quantum yields was performed for MeCN solutions of known concentration (2.5 mL volume, 10 mm pathlength cuvette) under irradiation with the aforementioned blue LED excitation source, the photon flux density of which was determined to be 2.01 × 10^–5^ einstein s^–1^ dm^–3^ through use of a K_3_Fe(C_2_O_4_)_3_·3H_2_O chemical actinometer. Photorelease quantum yield calculations were performed using GNU Octave software (version 6.2.0), freely available at https://www.gnu.org/software/octave/, using the method of Slep and co-workers.^72^

### Transient Absorption Spectroscopy

Spectra were recorded using a broadband ultrafast pump-probe transient absorption spectrometer “Helios” (Ultrafast Systems LLC), collecting data over a 3 ns time window with a time resolution of approximately 250 fs. A Ti:Sapphire amplifier system (Newport Spectra-Physics, Solstice Ace) producing 800 nm pulses at 1 kHz with 100 fs pulse duration was used to generate the probe beam and to also pump a TOPAS Prime OPA with associated near-infrared (NIR)–UV–vis unit to generate the excitation beam. The probe beam consisted of a white light continuum generated in a CaF_2_ crystal. Absorbance changes were monitored between 330 and 650 nm. Samples were excited with 0.5 μJ pulses at 285 nm, contained within a 0.2 cm pathlength quartz cuvette that was magnetically stirred during the measurements. Before data analysis, pre-excitation data was subtracted, and spectral chirp was corrected for. Kinetics were analyzed at the wavelengths of the highest intensity transient and bleach features. These traces were fitted with multiexponential functions with shared lifetime parameters.

### Single-Crystal X-ray Diffraction

X-ray diffraction data for **5** were collected at 150 K on a Bruker D8 Venture diffractometer equipped with a graphite monochromated Mo(Kα) radiation source and a cold stream of N_2_ gas. Solutions were generated by conventional Patterson heavy atom or direct methods and refined by full-matrix least-squared on *F*^2^ data, using SHELXS-97 and SHELXL software, respectively.^92^ Absorption corrections were applied based upon multiple and symmetry-equivalent measurements using SADABS.^93^ One of the hexafluorophosphate counterions displayed some rotational disorder, and this was refined over two positions using the *PART* instruction in the l.s. refinement with the disordered fluorine atoms restrained using the SIMU and DELU instructions. Crystallographic data are available as Supporting Information or can be downloaded from the Cambridge Crystallographic Data Centre.

Crystal data for CCDC 2162566, C_37_H_33_F_12_N_9_P_2_Ru, *M* = 994.73, monoclinic, *a* = 11.1689(7) Å, *b* = 27.7410(18) Å, *c* = 13.6303(8) Å, α = 90, β = 113.639(2), γ = 90, *V* = 3868.8(4) Å^3^, *T* = 150 K, space group *P*2_1_/*n*, *Z* = 4, 11 311 reflections measured, 8732 independent reflections (*R*_int_ = 0.0453). The final *R*_1_ values were 0.0515 (*I* > 2σ(*I*)). The final w**R**(*F*^2^) values were 0.1099 (*I* > 2σ(*I*)). The final *R*_1_ values were 0.0765 (all data). The final w**R**(*F*^2^) = 0.1193 (all data). The goodness of fit on *F*^2^ was 1.047. Largest peak and hole (e Å^–3^) 1.462/–0.699.

### Computational Details

The geometries of the ground states of complexes **1** to **6** were optimized using density functional theory using the B3LYP hybrid functional^94,95^ as implemented in the Orca 4.2.1 software package.^96,97^ Def2-ECP effective core potential and def2/j auxiliary basis set were used for ruthenium, with def2-tzvp(-f) basis sets used for all other atoms.^98^ All calculations were conducted using Grimme’s D3-BJ dispersion correction,^99,100^ along with the SMD implicit solvation model (acetonitrile).^101^ In these DFT calculations, the resolution-of-identity (RI) approximation for hybrid functionals (as implemented in ORCA) was employed to calculate the Coulomb energy term using the Ahlrichs/Weigend Def2-TZV basis as the auxiliary basis set and the exchange term by the so-called “chain-of-spheres exchange” (COSX) algorithm. For complexes **4** to **6**, the benzyl substituents of the triazole rings were replaced by methyl groups, as these will have little impact on the photophysical properties and also saves on computational expense. The ^3^MLCT states of the complexes were optimized by unrestricted DFT starting from the ground-state geometries, whereas ^3^MC_trans_ and ^3^MC_cis_ states were optimized from initial guess geometries, whose key bond lengths and angles were informed by previous data on related complexes.^59,60^ Molecular orbitals were visualized using the Gabedit software package with isosurfaces set to 0.02.

#### Synthesis of 4-(2-Methylpyrid-6-yl)-1,2,3-triazole (mpytz)

2-Methyl-6-(trimethylsilylethynyl)pyridine^102,103^ (466 mg, 2.46 mmol), benzyl azide (362 mg, 2.72 mmol, 1.1 equiv), sodium ascorbate (244 mg, 1.23 mmol, 0.5 equiv), CuSO_4_·5H_2_O (154 mg, 0.62 mmol, 0.25 equiv), and K_2_CO_3_ (467 mg, 3.38 mmol, 1.3 equiv) were added to a solvent mixture consisting of H_2_O (25 mL), THF (25 mL), ^*t*^BuOH (25 mL), and pyridine (5 mL). The reaction mixture was stirred at room temperature for 12 h. CH_2_Cl_2_ (75 mL), followed by conc. aq. NH_3_ (10 mL) was added, and the mixture was stirred vigorously at room temperature for a further 30 min. The organic phase was separated, and the remaining aqueous phase was extracted with a further 100 mL portion of CH_2_Cl_2_. The combined organic layers were washed successively with dilute aq. NH_3_ (3 × 100 mL), H_2_O (100 mL) and sat. brine (2 × 200 mL). The organic phase was dried over MgSO_4_ and filtered, and the solvent was removed in vacuo. The crude product was purified by column chromatography (SiO_2_, 1% MeOH/CH_2_Cl_2_), affording the product as a white solid. Yield = 521 mg, 85% ^1^H NMR (400 MHz, CDCl_3_) δ: 2.50 (s, 3H), 5.54 (s, 2H), 7.03 (d, *J* = 7.6 Hz, 1H), 7.27–7.38 (m, 5H), 7.60 (t, *J* = 7.6 Hz, 1H), 7.94 (d, *J* = 7.6 Hz, 1H), 8.04 (s, 1H). ^13^C NMR (101 MHz, CDCl_3_) δ: 24.53, 54.33, 117.27, 121.92, 122.45, 128.25, 128.79, 129.15, 134.59, 137.04, 149.10, 149.67, 158.23. HRMS (ESI) calcd for C_15_H_15_N_4_ ([M + H]^+^) 251.1291, found 251.1294.

#### Synthesis of [Ru(bpy)_2_(mbpy)](PF_6_)_2_ (**2**)

[Ru(bpy)_2_Cl_2_] (320 mg 0.66 mmol) was dissolved in ethanol (30 mL) and combined with mbpy (110 mg 0.66 mmol, 1 equiv). The solution was heated to 80 °C overnight under an N_2_ atmosphere in the dark. The solution was then cooled to room temperature, and an excess of NH_4_PF_6_ (323 mg 1.98 mmol), along with ethanol (30 mL), was added. The resultant red precipitate was collected by filtration, recrystallized from MeCN/Et_2_O, and purified further via column chromatography (SiO_2_, 10:1:1 (v/v/v) MeCN/H_2_O/KNO_3_ (aq.)). Subsequent counterion metathesis yielded the bright orange/red colored product as its hexafluorophosphate salt. Yield = 0.185 g, 32%. ^1^H NMR (400 MHz, CD_3_CN): δ 1.87 (s, 3H), 7.27–7.35 (m, 3H), 7.35–7.46 (m, 3H), 7.47–7.55 (m, 2H), 7.61 (d, *J* = 5.6 Hz, 1H), 7.75 (d, *J* = 5.6, 1H), 7.92–8.12 (m, 7H), 8.37 (d, *J* = 8.0 Hz, 1H), 8.41–8.55 (m, 5H). ^13^C NMR (101 MHz, CD_3_CN): δ 26.40, 122.83, 125.21, 125.35, 125.39, 125.47, 125.60, 127.98, 128.37, 128.39, 128.58, 128.83, 129.44, 138.45, 138.58, 138.71, 138.74, 138.98, 139.16, 152.10, 152.26, 152.35, 152.61, 154.05, 157.61, 157.98, 158.11, 158.21, 158.26, 159.23, 166.23. HRMS (ESI) calcd for C_31_H_26_N_6_RuPF_6_ ([M][PF_6_]^+^): 729.0898, found 729.0902; calcd for C_31_H_26_N_6_Ru (M^2+^): 292.0625, found 292.0632.

#### Synthesis of [Ru(bpy)_2_(mpytz)](PF_6_)_2_ (**5**)

Ru(bpy)_2_Cl_2_ (204 mg, 0.42 mmol) and mpytz (120 mg, 0.48 mmol, 1.1 equiv) were added to ethylene glycol (6 mL) and heated to 150 °C for 17 h in the dark under an N_2_ atmosphere. The resulting red/orange colored mixture was cooled to r.t. before the addition of an aqueous solution of NH_4_PF_6_ (703 mg, 4.31 mmol, 25 mL), which resulted in the formation of a bright orange colored precipitate. The mixture was stirred at room temperature for a further 10 min, and the solids were collected by filtration, being washed with H_2_O (30 mL), followed by Et_2_O (30 mL). The solids were redissolved in the minimum volume of MeCN and filtered, and the product was reprecipitated through the addition of excess Et_2_O. The precipitate was collected by filtration, washed with Et_2_O, and dried in vacuo, affording the title complex as a bright red/orange colored powder. NMR data indicate the presence of the photolysis product of the complex as a very minor contaminant. Yield = 376 mg, 94%. ^1^H NMR (400 MHz, CD_3_CN) δ: 1.84 (s, 3H), 5.45 (d, *J* = 15.4 Hz, 1H), 5.49 (d, *J* = 15.4 Hz, 1H), 7.14 (d, *J* = 7.2 Hz, 2H), 7.23–7.29 (m, 2H), 7.31–7.40 (m, 4H), 7.41–7.50 (m, 3H), 7.77 (d, *J* = 5.7 Hz, 1H), 7.83–7.94 (m, 3H), 7.95–8.02 (m, 2H), 8.05–8.13 (m, 3H), 8.36 (d, *J* = 8.05 Hz, 1H), 8.44 (d, *J* = 8.2 Hz, 1H), 8.49 (d, *J* = 8.2 Hz, 1H), 8.54 (d, *J* = 8.2 Hz, 1H), 8.60 (s, 1H). ^13^C NMR (101 MHz, CD_3_CN) δ: 26.02, 56.30, 121.16, 124.30, 124.82, 125.22, 125.24, 126.62, 127.66, 127.76, 128.27, 128.29, 128.43, 129.10, 129.86, 129.93, 134.53, 138.29, 138.52, 138.65, 139.52, 149.54, 151.63, 152.56, 152.81, 153.06, 154.04, 157.92, 158.17, 158.23, 158.92, 165.93. HRMS (ESI) calcd for C_35_H_30_N_8_RuPF_6_ ([M][PF_6_]^+^): 809.1273, found 809.1268, calcd for C_35_H_30_N_8_Ru (M^2+^): 332.0812, found 332.0815.

## Supporting Information Available

NMR and UV–vis absorption spectra recorded during photolysis of complexes, transient absorption spectra, and data from DFT calculations; optimized geometries for ground, MLCT, and MC states (PDF)
