# Supplementary material for: Not All 3MC States Are the Same: The Role of 3MCcis States in the Photochemical N∧N Ligand Release from [Ru(bpy)2(N∧N)]2+ Complexes
Source: Inorg Chem. 2022 Nov 30;61(49):19907–24. doi: 10.1021/acs.inorgchem.2c03146 (PMC9749030; doi:10.1021/acs.inorgchem.2c03146)
Supplement: Supplementary file 1 — ic2c03146_si_001.pdf [file ic2c03146_si_001.pdf]

Supporting Information

Not all ^{3}MC states are the same: the role of ^{3}MC$_{cis}$ states in the photochemical N^N ligand release from $[Ru(bpy)_2(N^\wedge N)]^{2+}$ complexes

Katie Eastham,[a] Paul A. Scattergood,*[a,b] Danny Chu,[a] Rayhaan Z. Boota,[a,b] Adrien Soupart,[d] Fabienne Alary,[d] Isabelle M. Dixon,[d] Craig R. Rice,[a] Samantha Hardman[c] & Paul I.P. Elliott*[a,b]

[a] *Department of Chemistry, University of Huddersfield, Queensgate, Huddersfield, HD1 3DH, UK*

[b] *Centre for Functional Materials, University of Huddersfield, Queensgate, Huddersfield, HD1 3DH, UK*

[c] *Manchester Institute of Biotechnology, The University of Manchester, 131 Princess Street, Manchester M1 7DN, UK*

[d] *Laboratoire de Chimie et Physique Quantiques, UMR 5626 CNRS/Université Toulouse 3 - Paul Sabatier, Université de Toulouse, 118 route de Narbonne, Toulouse, 31062, France*

* corresponding authors: p.i.elliott@hud.ac.uk; p.scattergood@hud.ac.uk

Contents

**Figure S1**	^{1}H NMR spectrum of the ligand mpytz	**S3**
**Figure S2**	^{13}C NMR spectrum of the ligand mpytz	**S3**
**Figure S3**	^{1}H NMR spectrum of $[Ru(bpy)_2(mbpy)](PF_6)_2$ (**2**)	**S4**
**Figure S4**	^{13}C NMR spectrum of $[Ru(bpy)_2(mbpy)](PF_6)_2$ (**2**)	**S4**
**Figure S5**	^{1}H NMR spectrum of $[Ru(bpy)_2(mpytz)](PF_6)_2$ (**5**)	**S5**
**Figure S6**	^{13}C NMR spectrum of $[Ru(bpy)_2(mpytz)](PF_6)_2$ (**5**)	**S5**
**Figure S7**	ps-Transient absorption spectra recorded for complex **1**	**S6**
**Figure S8**	ps-Transient absorption spectra recorded for complex **2**	**S6**
**Figure S9**	ps-Transient absorption spectra recorded for complex **3**	**S6**
**Figure S10**	ps-Transient absorption spectra recorded for complex **4**	**S7**
**Figure S11**	ps-Transient absorption spectra recorded for complex **6**	**S7**
**Figure S12**	UV-Visible absorption spectra recorded during the photolysis of **2**,**3** and **4**	**S8**
**Figure S13**	^{1}H NMR spectra recorded before and late in the photolysis of **2**	**S9**
**Figure S14**	^{1}H NMR spectra recorded before and at the end of the photolysis of **3**	**S9**
**Figure S15**	^{1}H NMR spectra recorded before and late in the photolysis of **4**	**S10**
**Figure S16**	^{1}H NMR spectra recorded before and late in the photolysis of **5**	**S10**
**Figure S17**	^{1}H NMR spectra recorded before, during and at the end of the photolysis of **6**	**S11**
**Figure S18**	^{1}H NMR spectrum of $[Ru(bpy)_2(MeCN)_2]^{2+}$	**S11**
**Table S1**	Calculated Ru-N bond lengths for ^{1}GS, ^{3}MLCT and ^{3}MC states of **1-6**	**S12**
**Figure 19**	Plots of singlet ground state HOMO, LUMO and dσ* orbitals for **2-5**	**S13**

Figure 20 Plot of the SONOs for the T_1 ^{3}MLCT states of complexes **2-5** **S13**

Figure S21 Plots of the SONOs for the optimised ^{3}MC states of complexes **2-4** **S14**

Figure S21 Cont. Plots of the SONOs for the optimised ^{3}MC states of complexes **5-6** **S15**

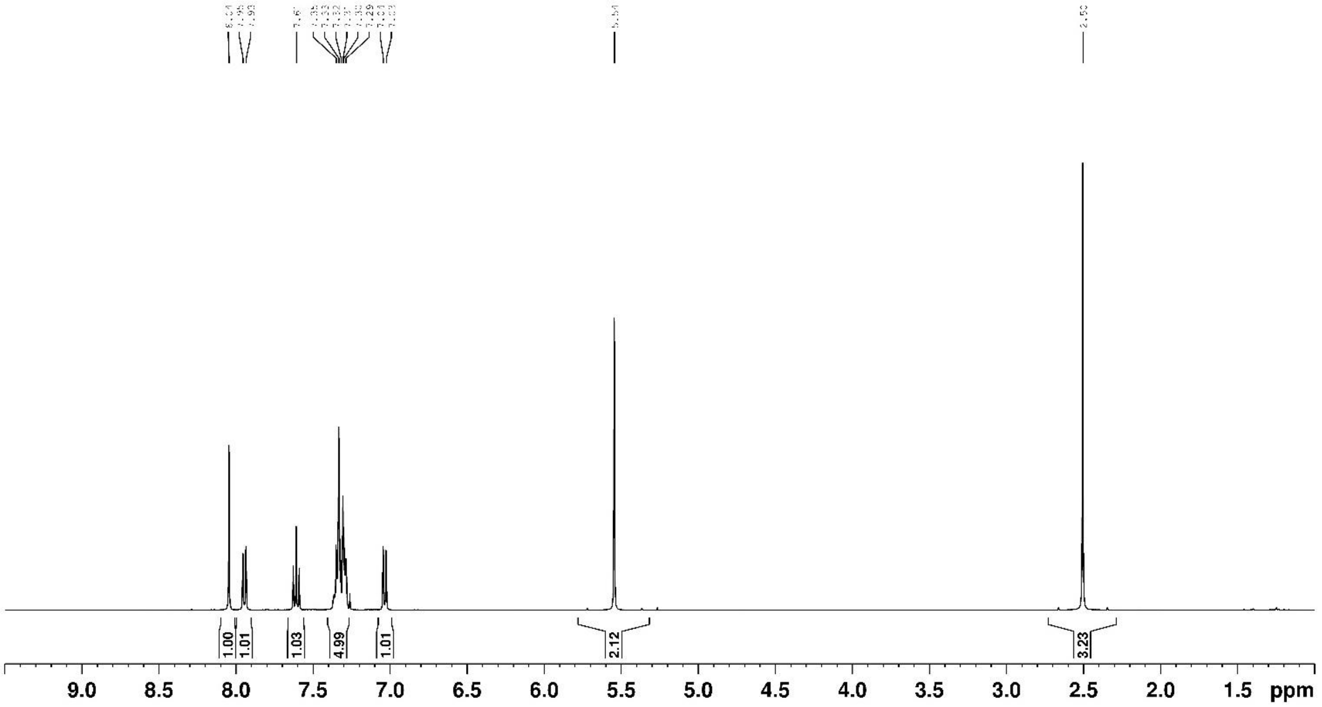

Figure S1. ^{1}H NMR spectrum of the ligand mpytz in $CDCl_3$.

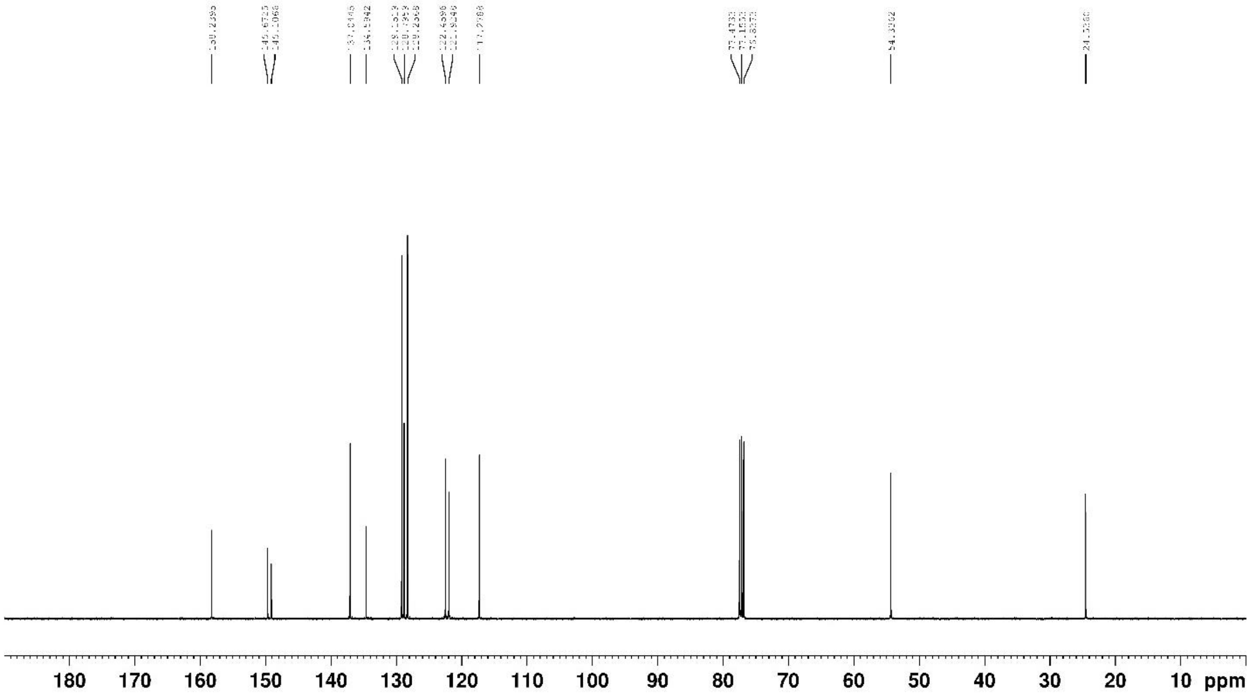

Figure S2. ^{13}C NMR spectrum of the ligand mpytz in $CDCl_3$.

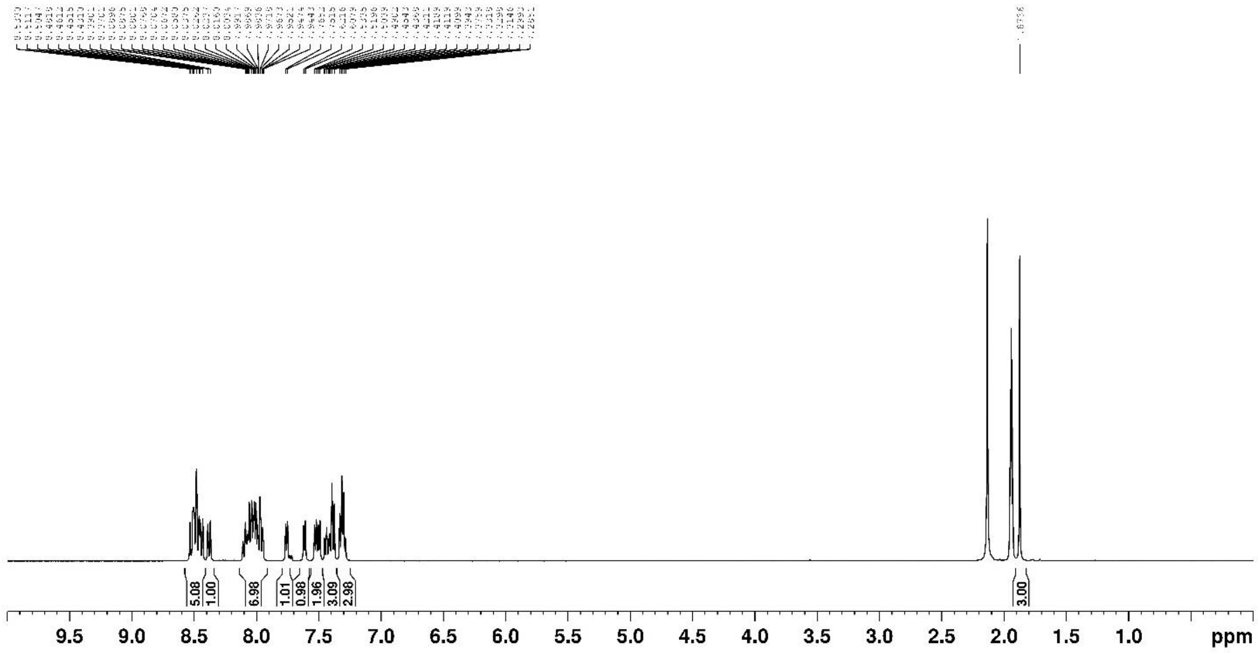

Figure S3. ^{1}H NMR spectrum of $[Ru(bpy)_2(mbpy)](PF_6)_2$ (**2**) in d_3-acetonitrile.

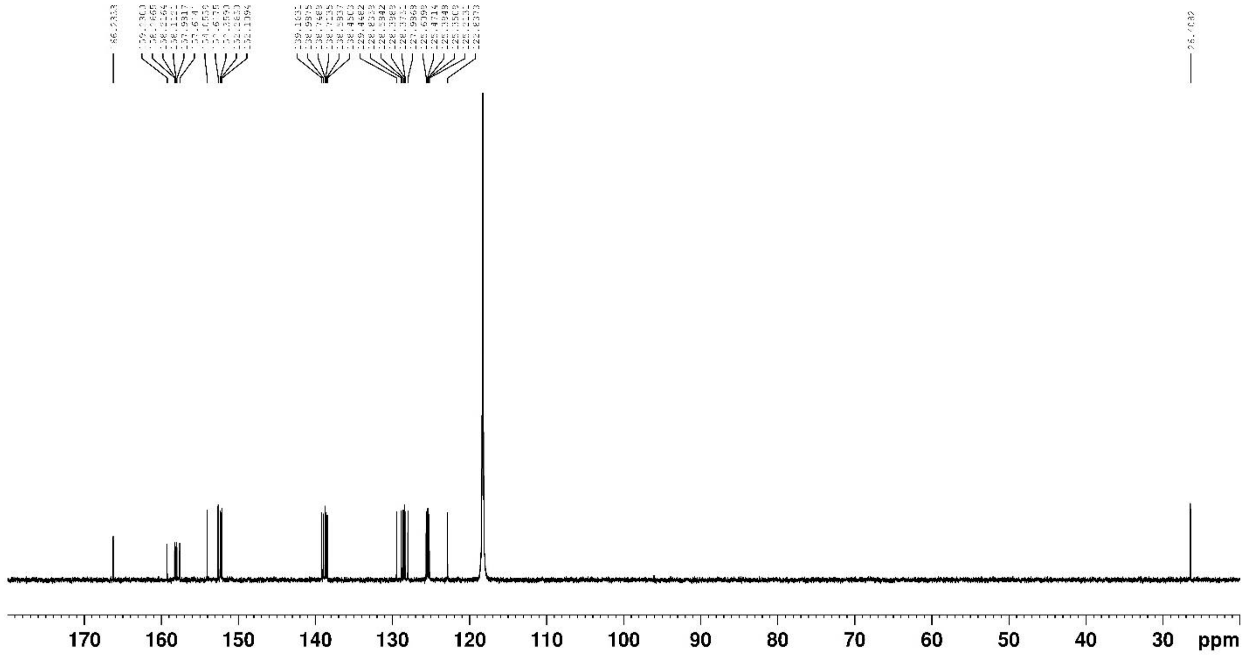

Figure S4. ^{13}C NMR spectrum of $[Ru(bpy)_2(mbpy)](PF_6)_2$ (**2**) in d_3-acetonitrile.

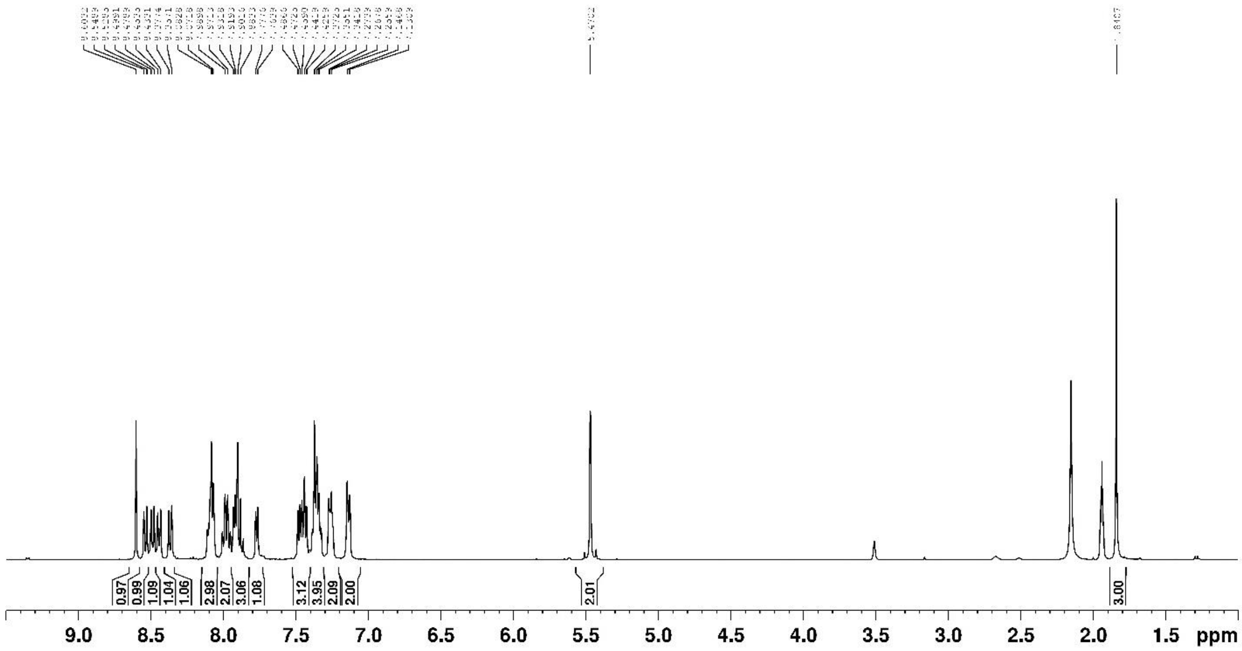

Figure S5. ^{1}H NMR spectrum of $[Ru(bpy)_2(mpytz)](PF_6)_2$ (**5**) in d_3-acetonitrile

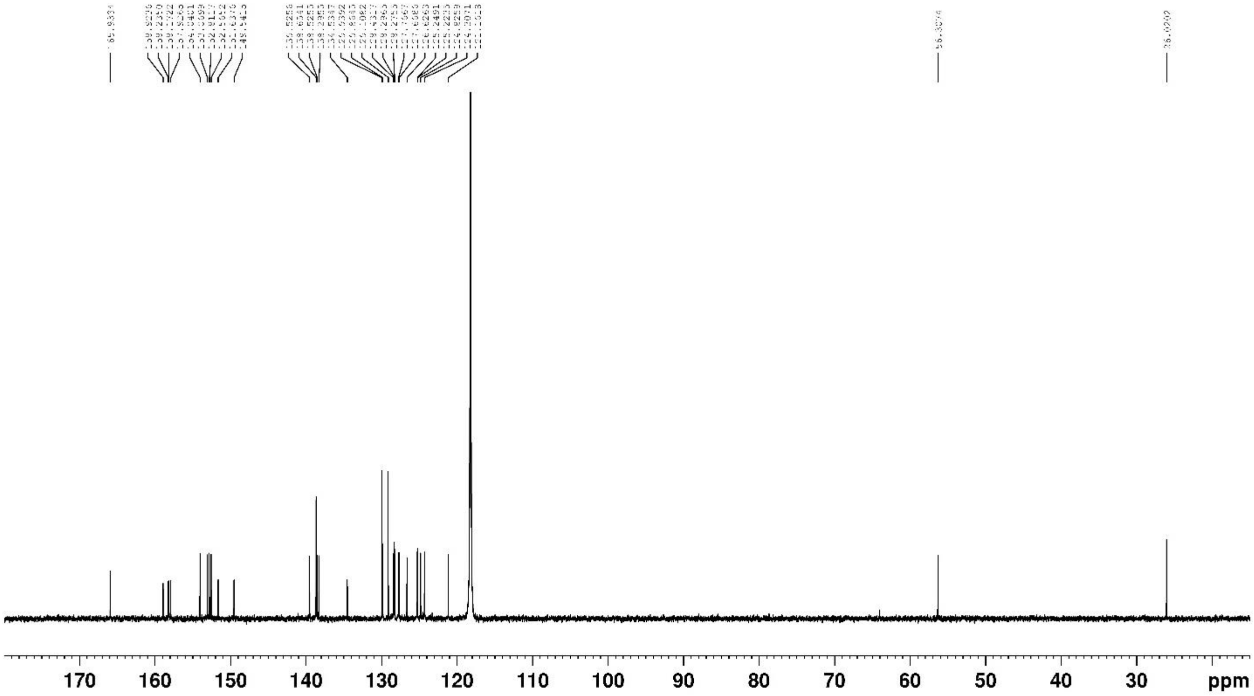

Figure S6. ^{13}C NMR spectrum of $[Ru(bpy)_2(mpytz)](PF_6)_2$ (**5**) in d_3-acetonitrile

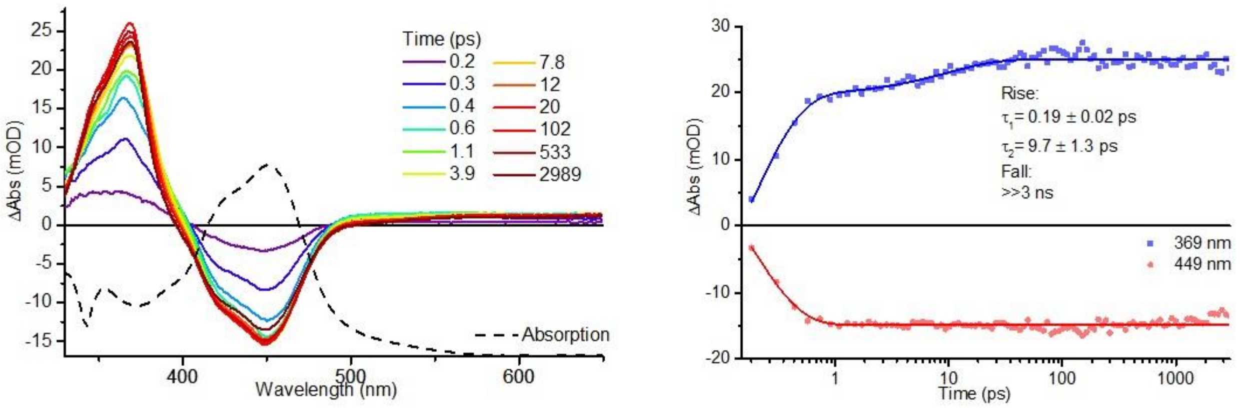

Figure S7. Left: ps-Transient absorption spectra recorded for **1** in acetonitrile (λ_{ex} = 285 nm), overlaid with the ground state electronic absorption profile (dashed line). Right: Selected single point kinetic traces and associated time constants obtained from global analysis.

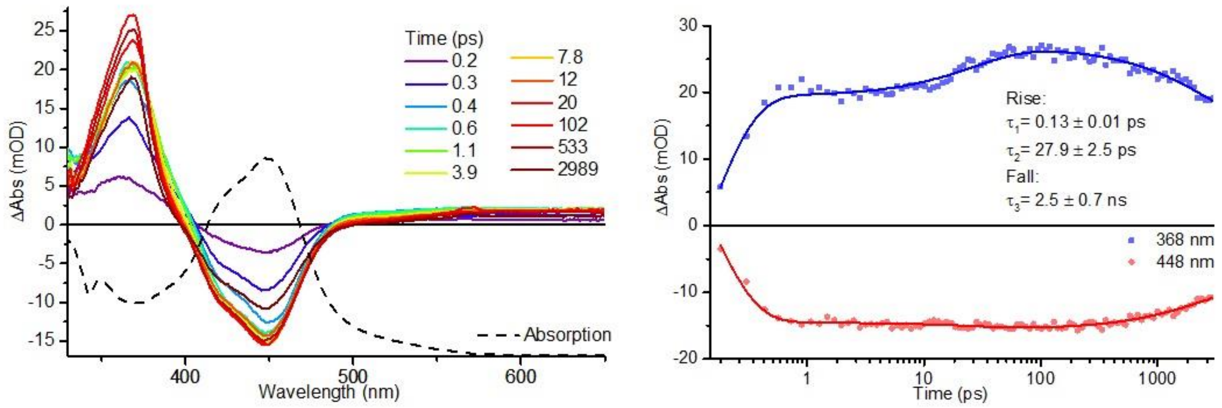

Figure S8. Left: ps-Transient absorption spectra recorded for **2** in acetonitrile (λ_{ex} = 285 nm), overlaid with the ground state electronic absorption profile (dashed line). Right: Selected single point kinetic traces and associated time constants obtained from global analysis.

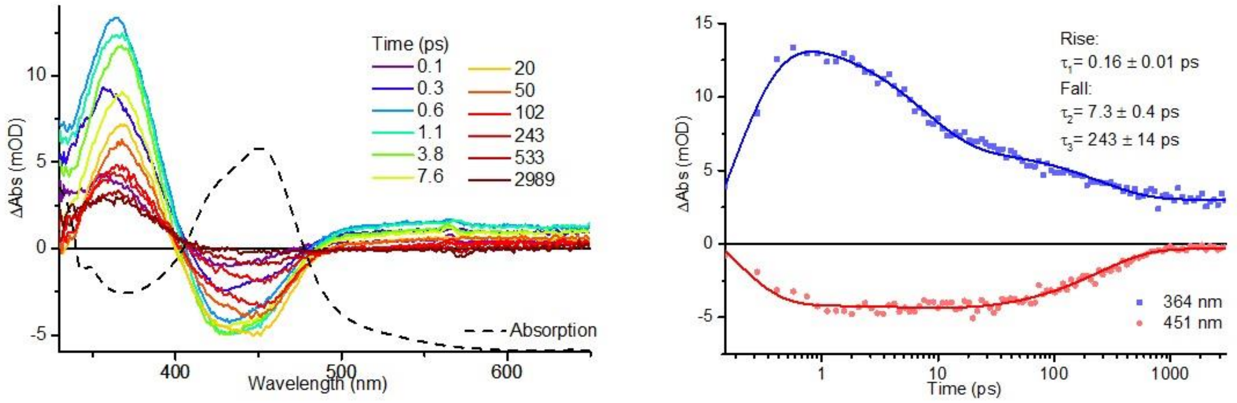

Figure S9. Left: ps-Transient absorption spectra recorded for **3** in acetonitrile (λ_{ex} = 285 nm), overlaid with the ground state electronic absorption profile (dashed line). Right: Selected single point kinetic traces and associated time constants obtained from global analysis. The transient feature at 364 nm does not appear to fully return in the 3 ns window of the experiment despite GSB recovery and is tentatively ascribed to possible photochemical decomposition.

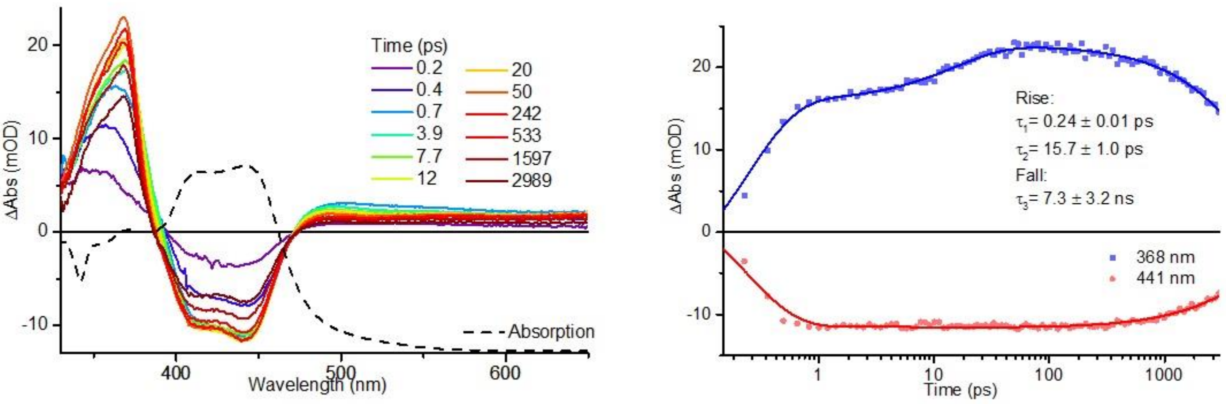

Figure S10. Left: ps-Transient absorption spectra recorded for **4** in acetonitrile (λ_{ex} = 285 nm), overlaid with the ground state electronic absorption profile (dashed line). Right: Selected single point kinetic traces and associated time constants obtained from global analysis.

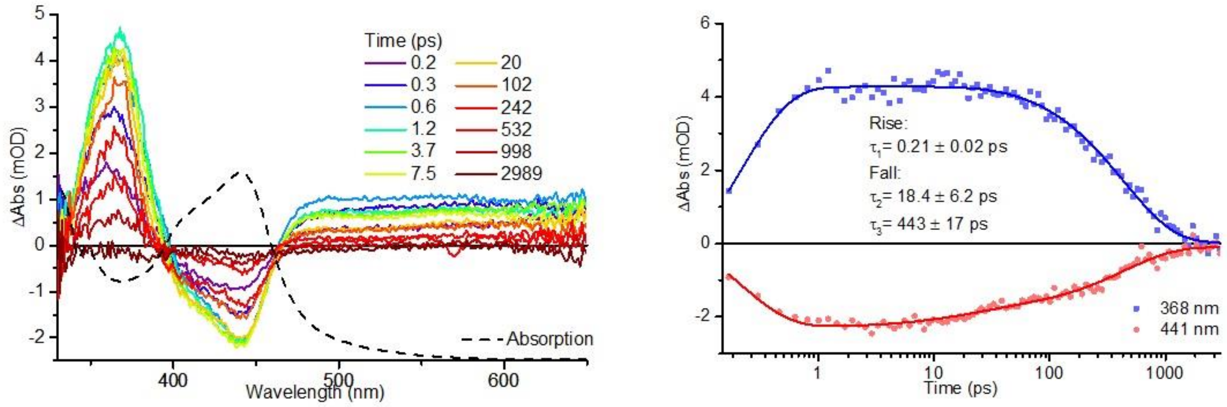

Figure S11. Left: ps-Transient absorption spectra recorded for **6** in acetonitrile (λ_{ex} = 285 nm), overlaid with the ground state electronic absorption profile (dashed line). Right: Selected single point kinetic traces and associated time constants obtained from global analysis.

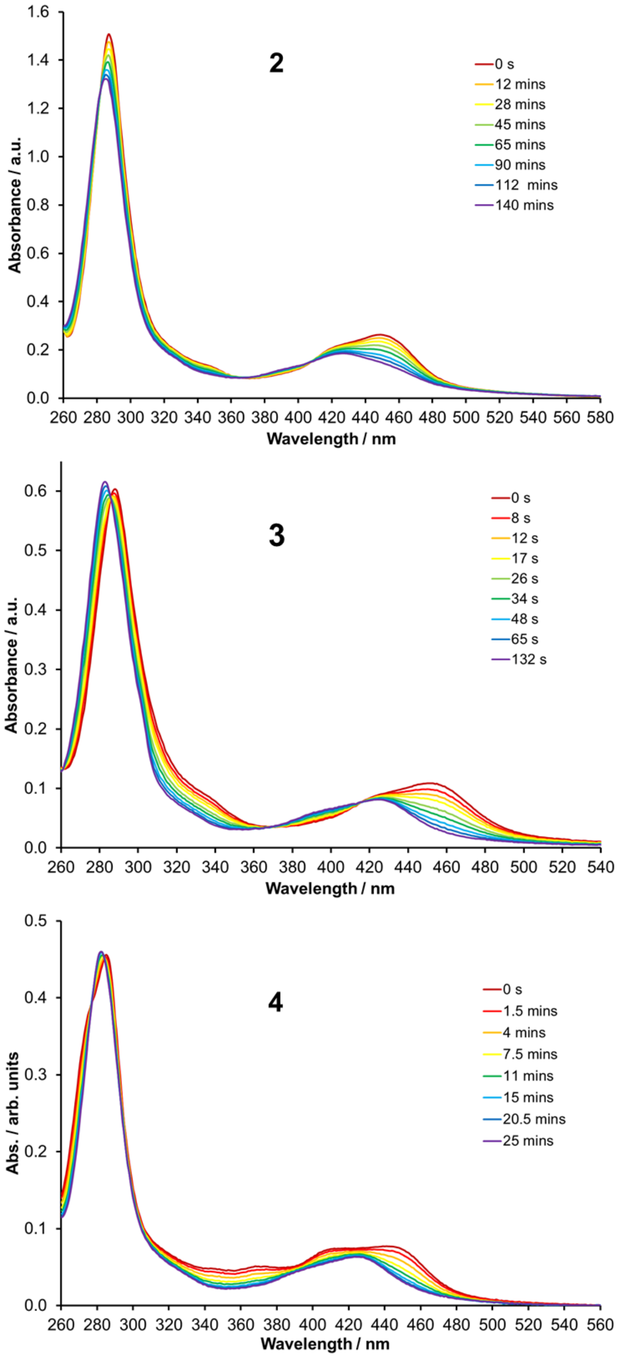

Figure S12. UV-visible absorption spectra of complexes **2**, **3** and **4** recorded during photolysis in acetonitrile (blue LED, 446 nm, 17 ± 1 mW).

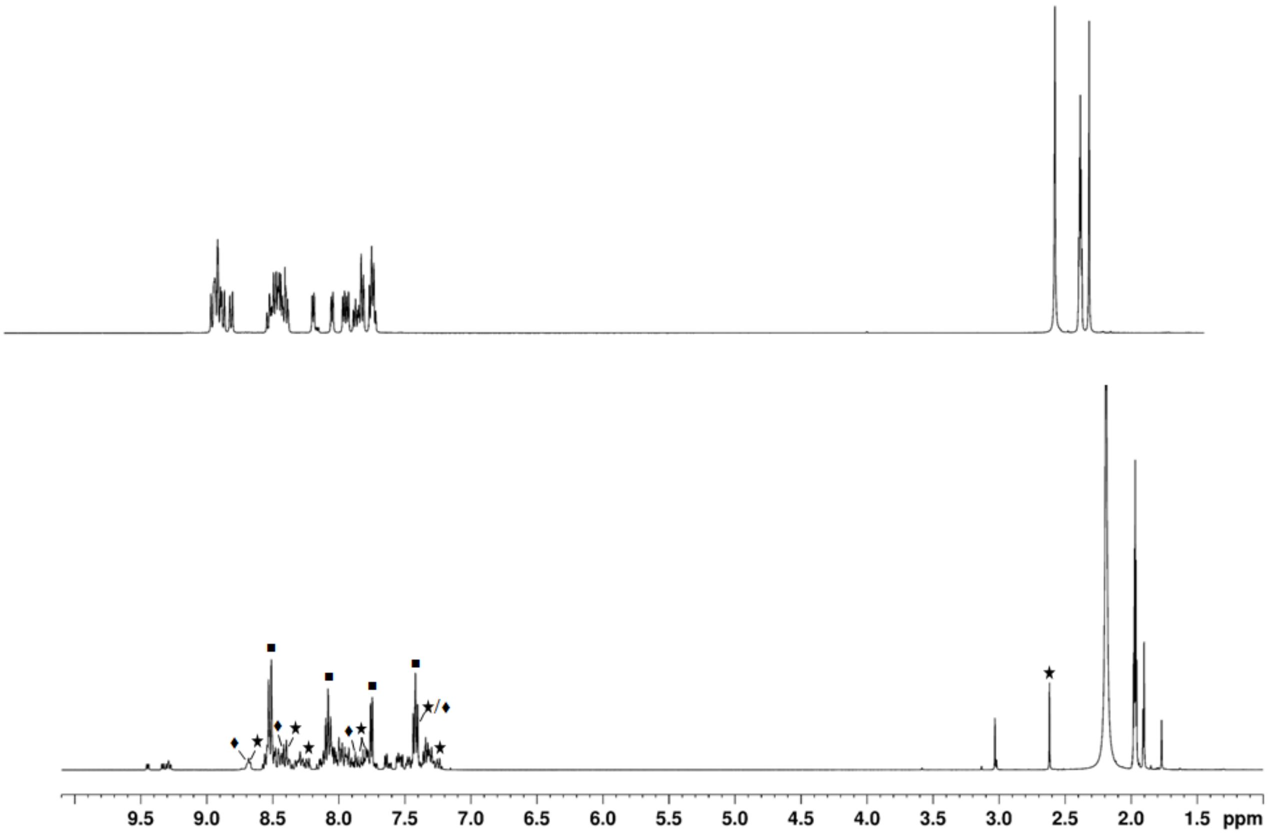

Figure S13. ^{1}H NMR spectra before (top) and late (bottom) in photolysis of $[Ru(bpy)_2(mbpy)]^{2+}$ (**2**) in d_3-acetonitrile (★ free mbpy ligand, ♦ free bpy ligand, ▪ $[Ru(bpy)_3]^{2+}$).

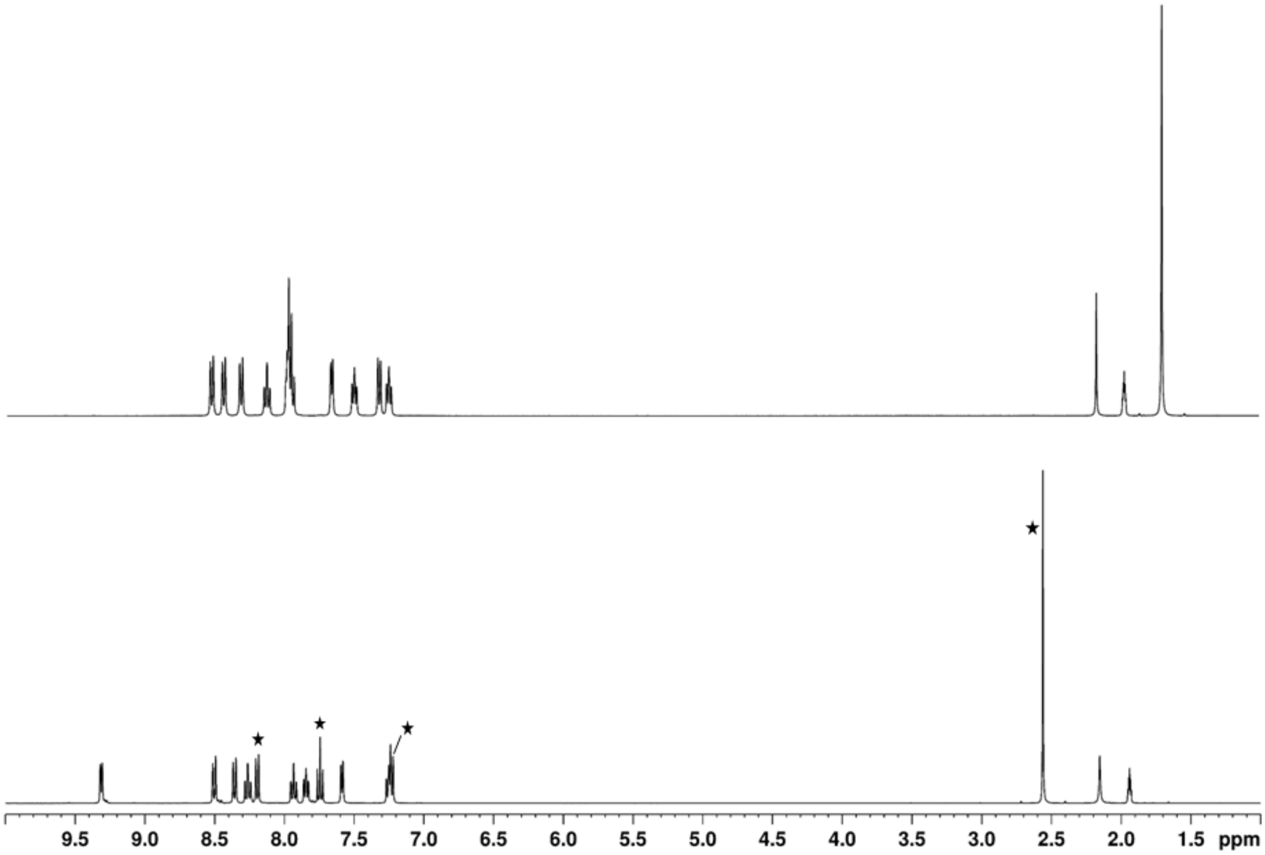

Figure S14. ^{1}H NMR spectra before (top) and at the end (bottom) of photolysis of $[Ru(bpy)_2(dmbpy)]^{2+}$ (**3**) in d_3-acetonitrile (★ free dmbpy ligand).

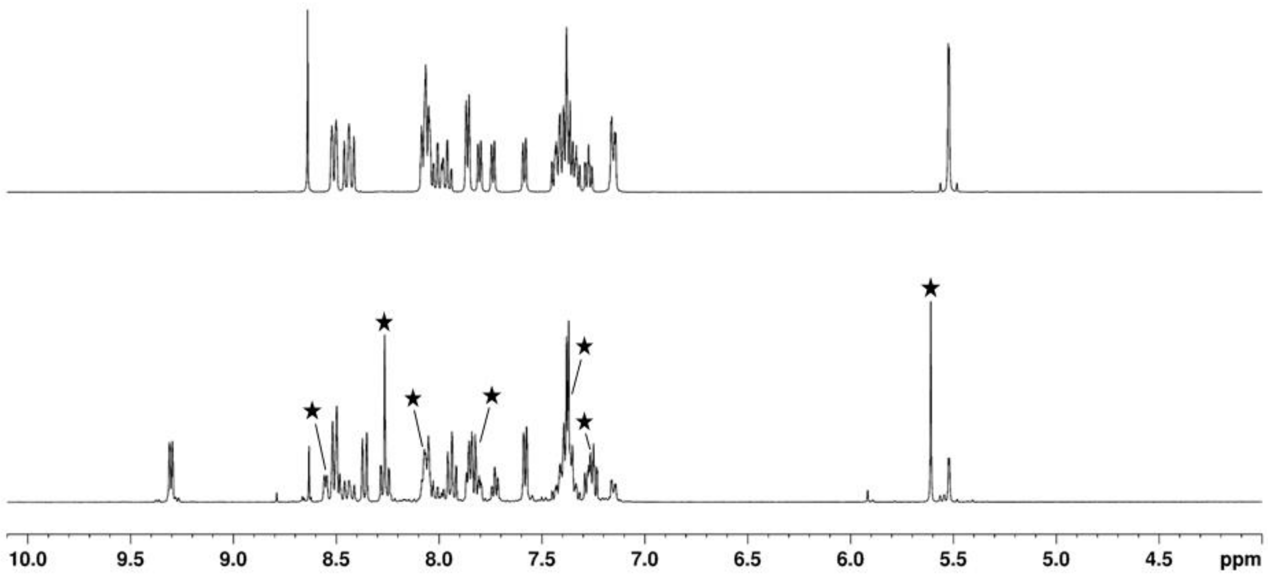

Figure S15. ^{1}H NMR spectra before (top) and late (bottom) in photolysis of $[Ru(bpy)_2(pytz)]^{2+}$ (**4**) in d_3-acetonitrile (★ free pytz ligand).

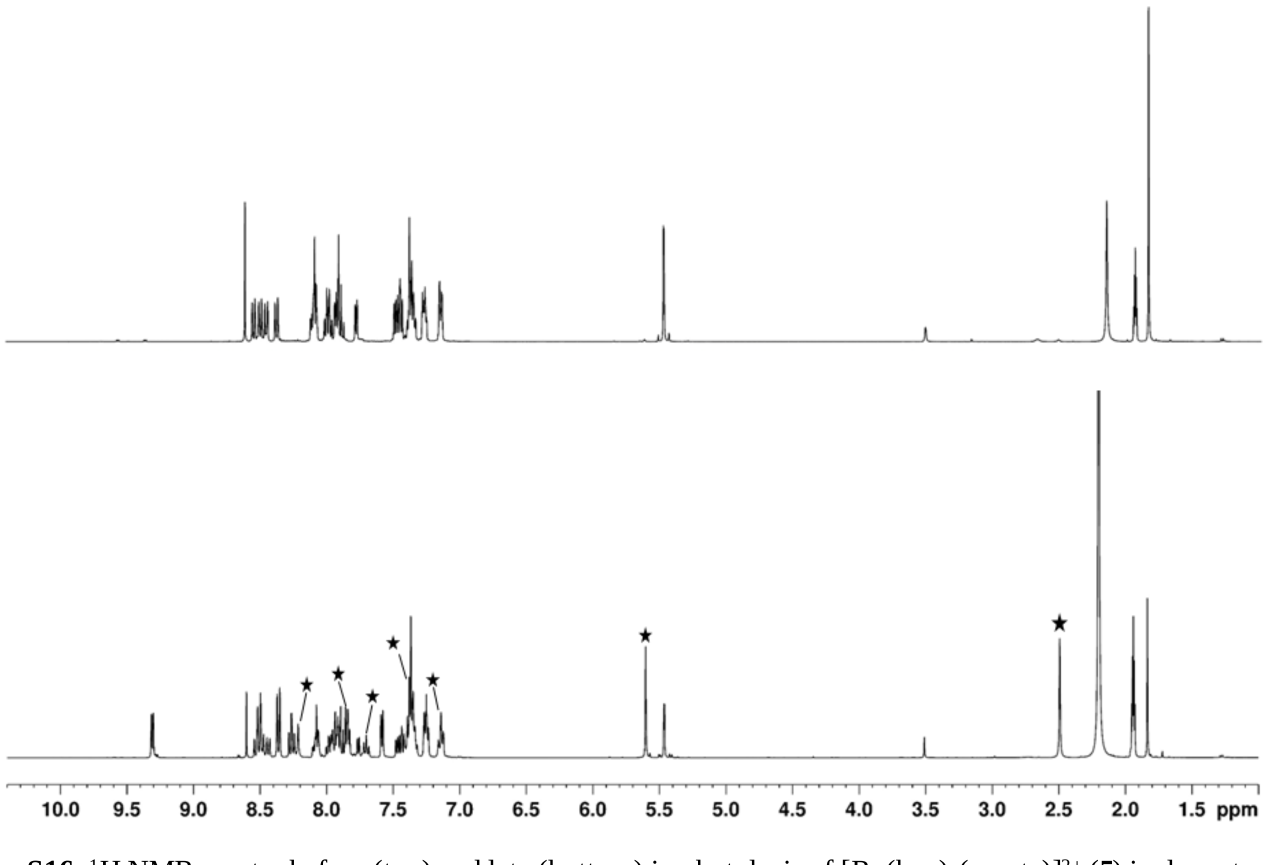

Figure S16. ^{1}H NMR spectra before (top) and late (bottom) in photolysis of $[Ru(bpy)_2(mpytz)]^{2+}$ (**5**) in d_3-acetonitrile (★ free mpytz ligand).

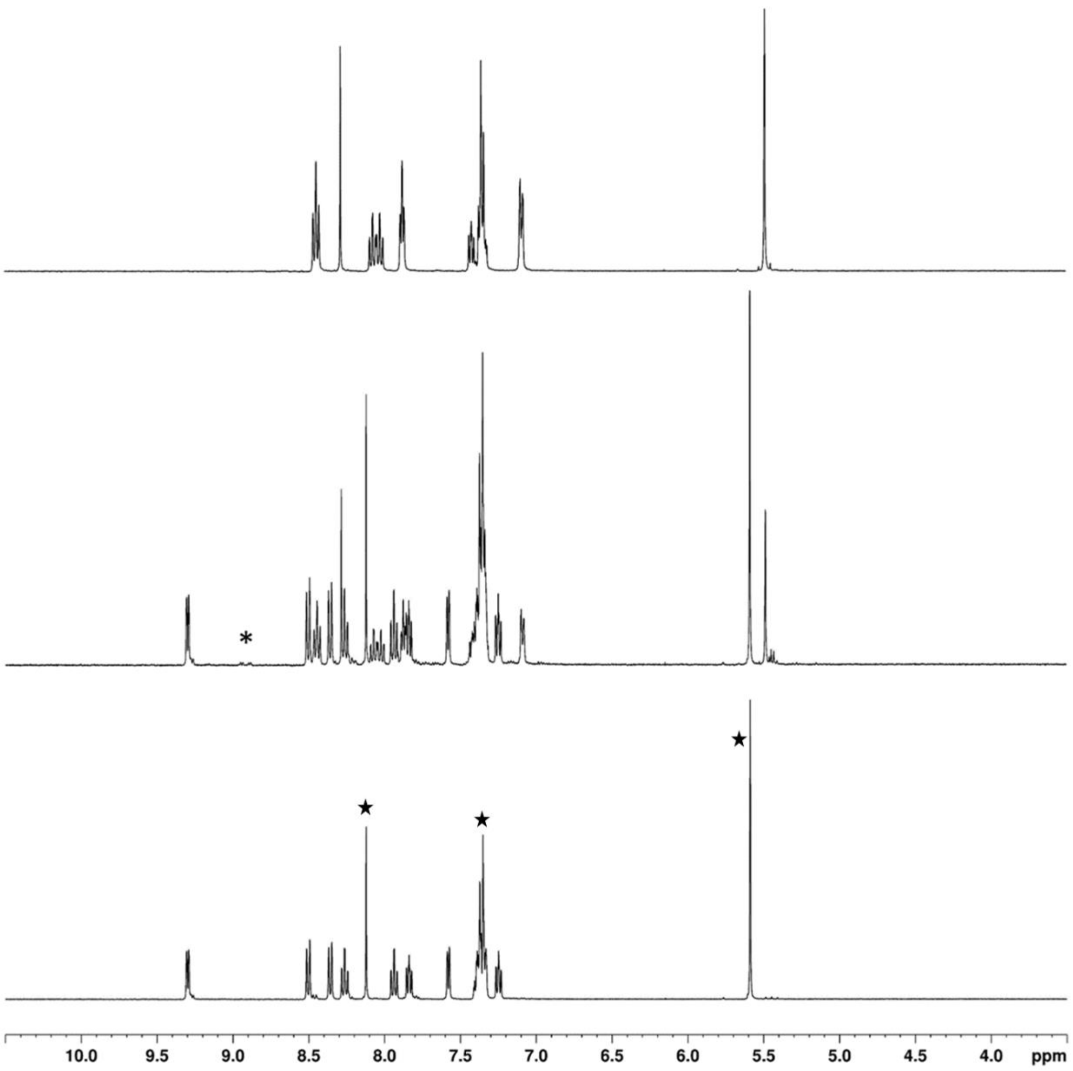

Figure S17. ^{1}H NMR spectra before (top), during (middle) and at the end (bottom) of photolysis of $[Ru(bpy)_2(btz)]^{2+}$ (**6**) in d_3-acetonitrile (* low concentration intermediate, ★ free btz).

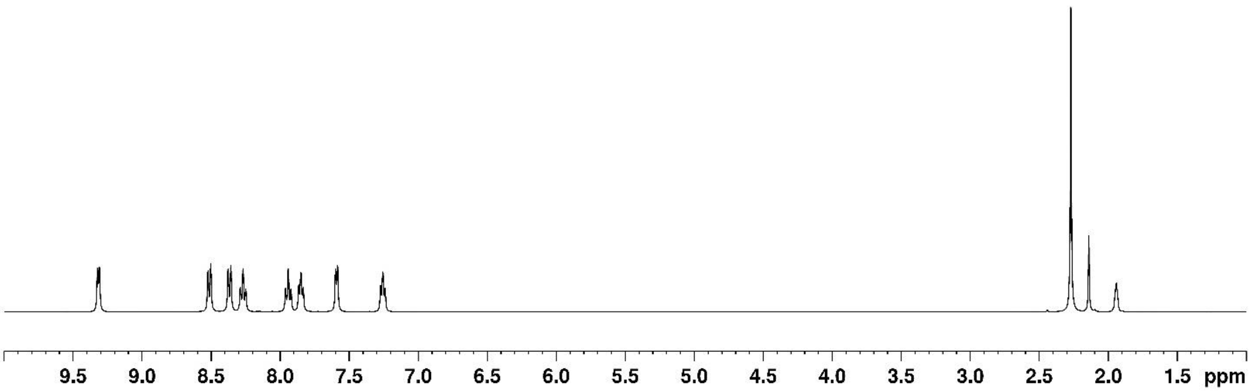

Figure S18. ^{1}H NMR spectrum of $[Ru(bpy)_2(MeCN)_2][PF_6]_2$ recorded in d_3-acetonitrile.

Table S1. Calculated Ru-N bond lengths (Å) for ^{1}GS, ^{3}MLCT and ^{3}MC states of complexes **1** to **6**. Bond lengths for ^{3}MC states in bold indicate principally elongated Ru-N bonds (refer to Figure 9 for definitions of A, B & C for ^{3}MC$_{trans}$).

1

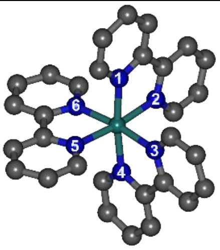

	^{1}GS	^{3}MLCT	^{3}MC$_{trans}$	^{3}MC$_{cis}$
Ru-N(1)	2.08	2.10	**2.53**	2.18
Ru-N(2)	2.08	2.08	2.14	2.08
Ru-N(3)	2.07	2.05	2.13	**2.38**
Ru-N(4)	2.07	2.05	**2.42**	**2.53**
Ru-N(5)	2.07	2.07	2.09	2.08
Ru-N(6)	2.07	2.09	2.07	2.13

2

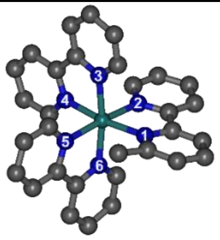

	^{1}GS	^{3}MLCT	^{3}MC$_{trans,A}$	^{3}MC$_{trans,B}$	^{3}MC$_{trans,C}$
Ru-N(1)	2.15	2.16	**2.52**	2.16	2.16
Ru-N(2)	2.07	2.07	2.13	**2.43**	2.07
Ru-N(3)	2.07	2.03	2.13	2.07	**2.45**
Ru-N(4)	2.07	2.05	**2.42**	2.10	2.14
Ru-N(5)	2.08	2.08	2.09	**2.47**	2.15
Ru-N(6)	2.08	2.10	2.08	2.17	**2.44**

3

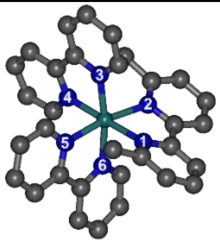

	^{1}GS	^{3}MLCT	^{3}MC$_{trans,A}$	^{3}MC$_{trans,B}$
Ru-N(1)	2.13	2.12	2.16	2.10
Ru-N(2)	2.13	2.13	**2.47**	2.15
Ru-N(3)	2.07	2.08	2.07	**2.35**
Ru-N(4)	2.07	2.07	2.09	2.11
Ru-N(5)	2.08	2.04	**2.51**	2.15
Ru-N(6)	2.09	2.07	2.16	**2.60**

4

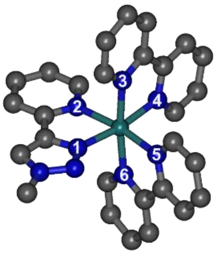

	^{1}GS	^{3}MLCT	^{3}MC$_{trans,A}$	^{3}MC$_{trans,B}$	^{3}MC$_{trans,C}$	^{3}MC$_{cis}$
Ru-N(1)	2.05	2.08	**2.52**	2.11	2.07	**2.28**
Ru-N(2)	2.11	2.10	2.14	**2.59**	2.11	**2.68**
Ru-N(3)	2.07	2.04	2.13	2.07	**2.47**	2.07
Ru-N(4)	2.07	2.04	**2.42**	2.07	2.12	2.10
Ru-N(5)	2.06	2.07	2.09	**2.39**	2.13	2.19
Ru-N(6)	2.07	2.09	2.08	2.12	**2.45**	2.08

5

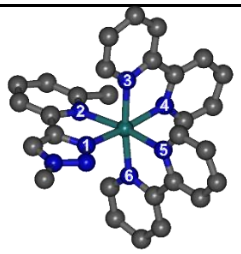

	^{1}GS	^{3}MLCT	^{3}MC$_{trans,A}$	^{3}MC$_{trans,B}$	^{3}MC$_{trans,C}$
Ru-N(1)	2.04	2.06	2.09	**2.51**	2.06
Ru-N(2)	2.17	2.17	**2.62**	2.17	2.18
Ru-N(3)	2.08	2.05	2.07	2.17	**2.48**
Ru-N(4)	2.08	2.05	2.07	**2.41**	2.13
Ru-N(5)	2.06	2.06	**2.38**	2.09	2.12
Ru-N(6)	2.06	2.07	2.13	2.07	**2.42**

6

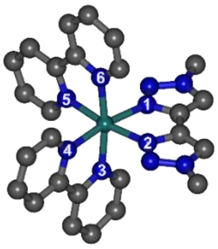

	^{1}GS	^{3}MLCT	^{3}MC$_{trans,A}$	^{3}MC$_{trans,B}$	^{3}MC$_{cis}$
Ru-N(1)	2.08	2.08	2.10	2.09	**2.29**
Ru-N(2)	2.08	2.10	**2.76**	2.09	**2.71**
Ru-N(3)	2.07	2.09	2.07	**2.45**	2.07
Ru-N(4)	2.06	2.07	2.07	2.13	2.10
Ru-N(5)	2.06	2.04	**2.32**	2.12	2.18
Ru-N(6)	2.08	2.04	2.13	**2.45**	2.09

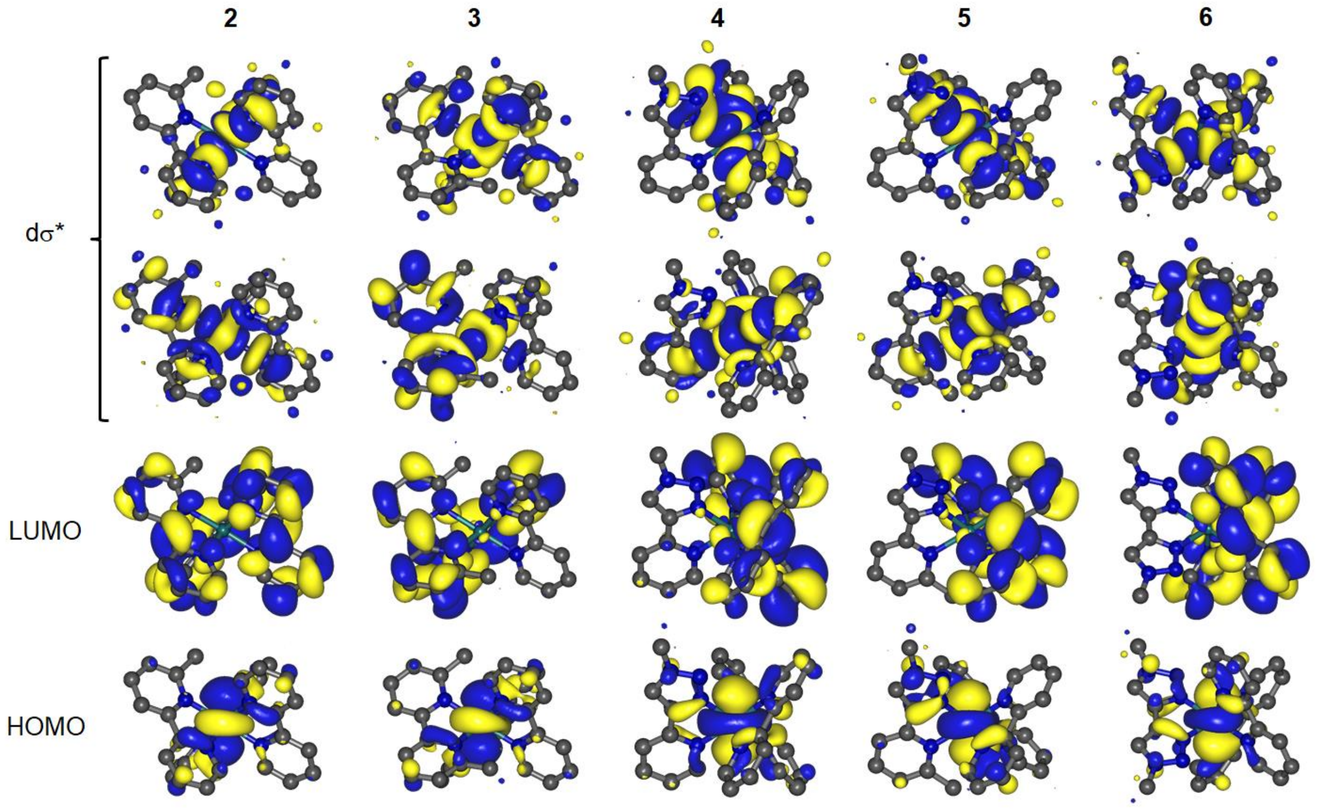

Figure S19. Plots of singlet ground state HOMO, LUMO and dσ* orbitals for complexes **2** to **5** (isosurfaces set at 0.02).

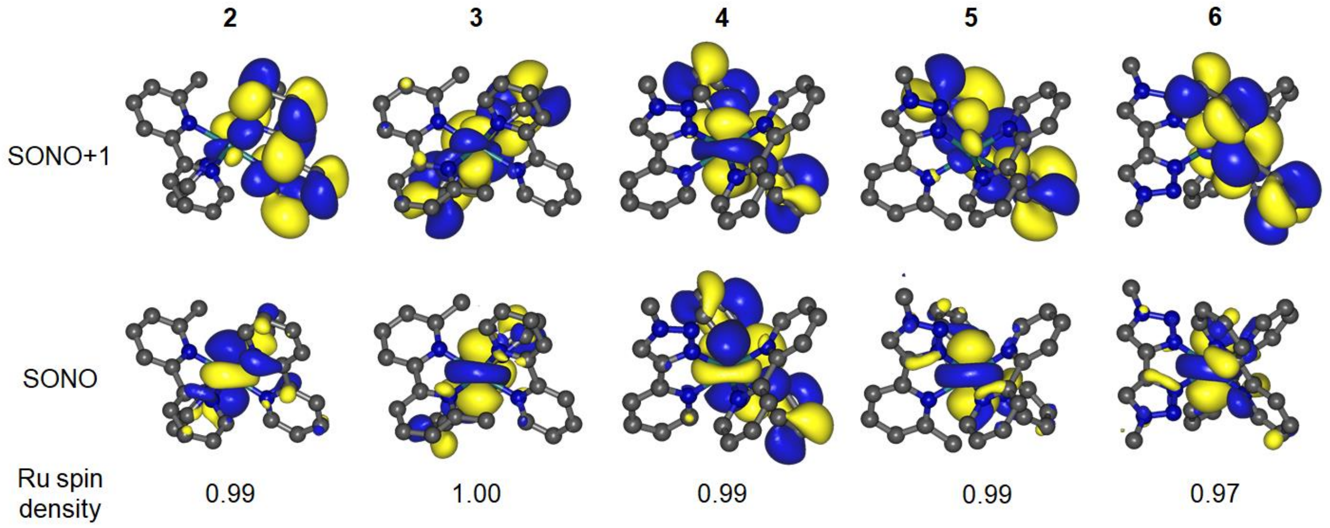

Figure S20. Plot of the singly occupied natural orbitals (SONOs) for the T_1 ^{3}MLCT states of complexes **2** to **5** (isosurfaces set at 0.02).

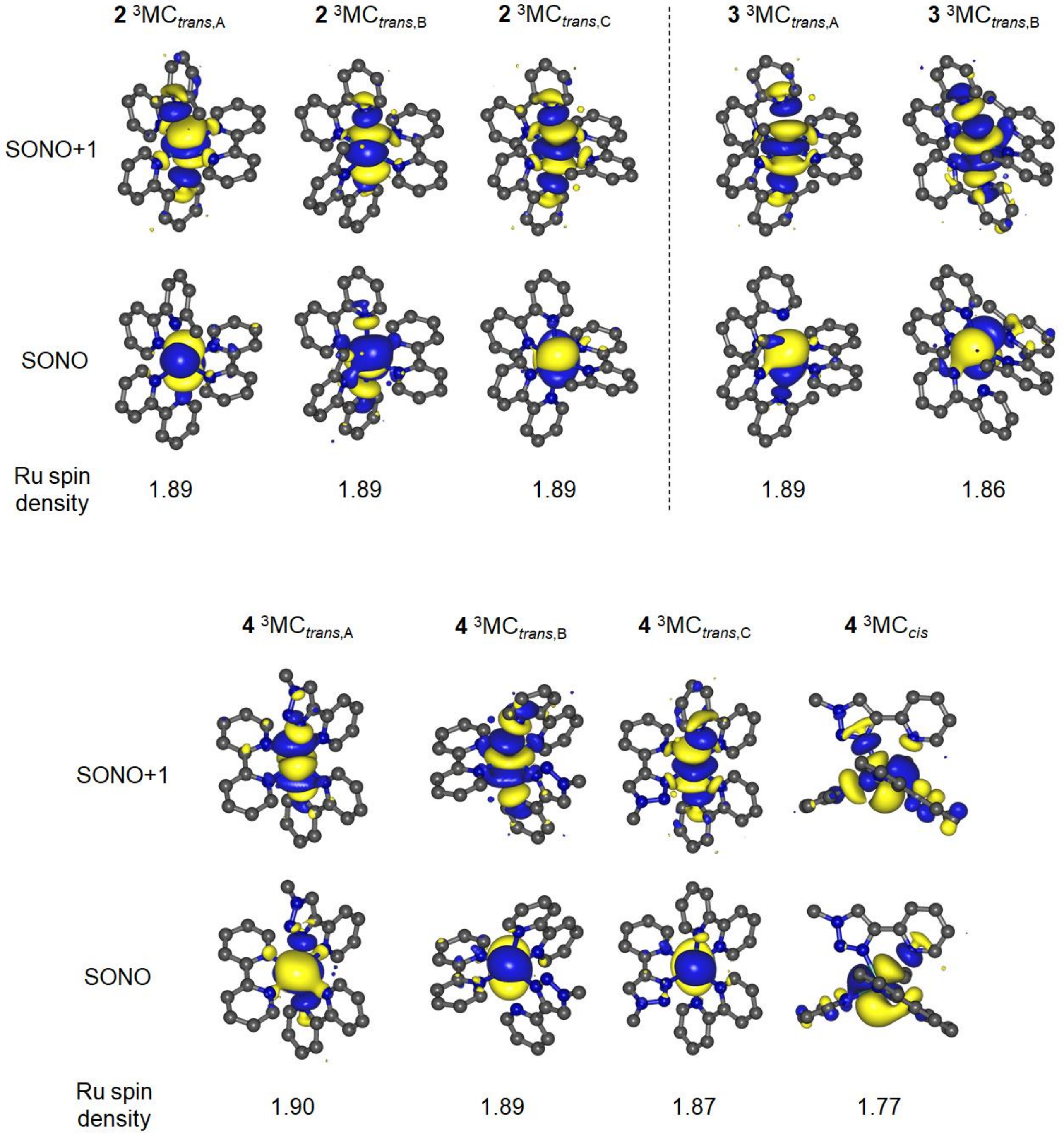

Figure S21. Plots of the singly occupied natural orbitals (SONOs) for the optimised ^{3}MC states of complexes **2** to **4** (isosurfaces set at 0.02).

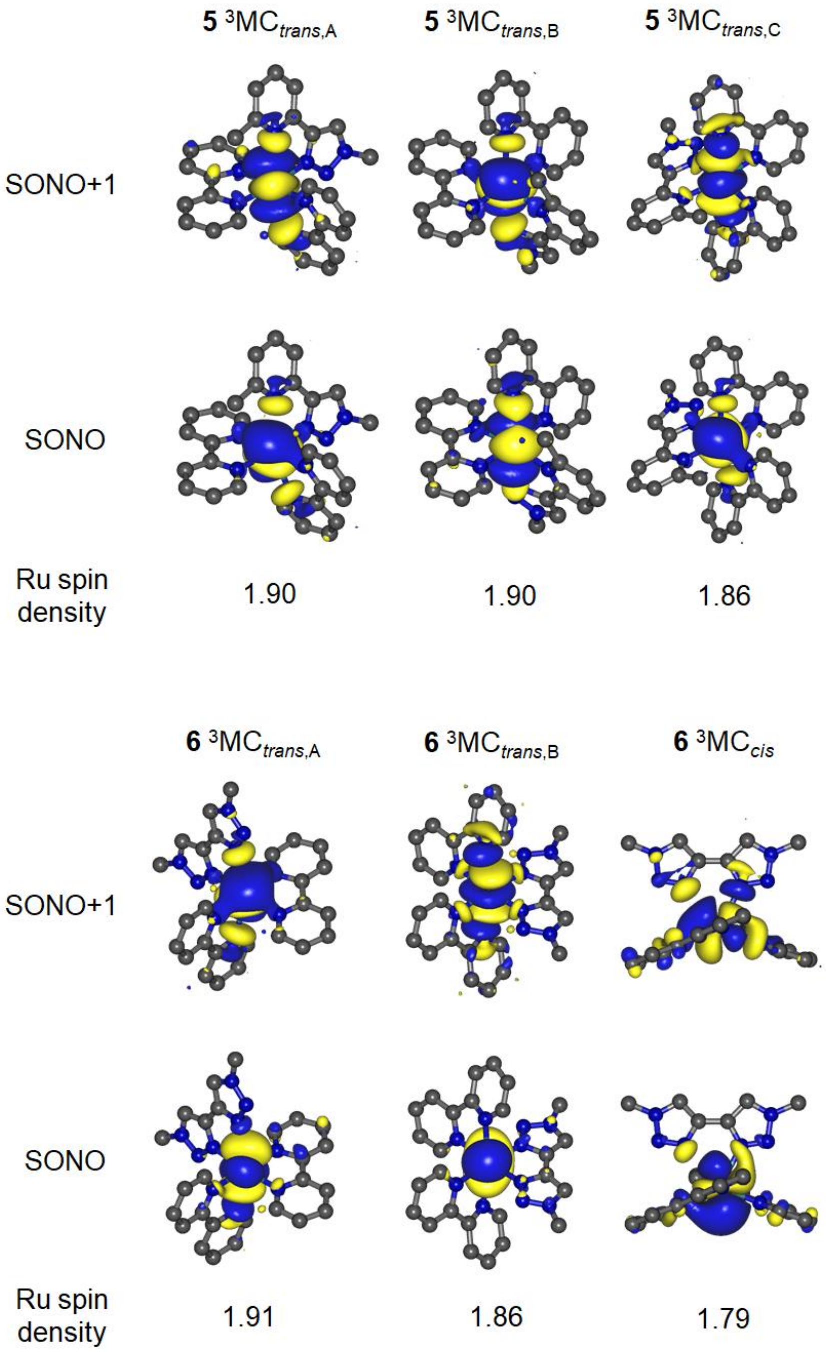

Figure S21 (continued). Plots of the singly occupied natural orbitals (SONOs) for the optimised ^{3}MC states of complexes **5** and **6** (isosurfaces set at 0.02).
